# *Plasmodium vivax* and *Plasmodium falciparum* mixed infections in human and mosquito hosts: The impact of multi-species infection on parasite densities and transmission to mosquitoes

**DOI:** 10.1371/journal.pntd.0014148

**Published:** 2026-07-24

**Authors:** Wakweya Chali, Legesse Alamerie Ejigu, Tigist Atele, Solomon Sisay, Gutema Jebessa, Getnet Habtamu, Melat Abdo, Amanuel Shimelash, Mulugeta Demisse, Migbaru Keffale Bezabih, Desalegn Nibret, Addisu Gizat, Kebede Getachew Beyene, Kassahun Abi, Ayalew Jejaw Zeleke, Banchayehu Getnet, Zewdu Selemon Amare, Teresa Beyena, Tirhas Endale, Meskele Dechasa, Fekadu Massebo, Fikregabrail Aberra Kassa, Sagni Challi, Hassen Mamo, Chris Drakeley, Teun Bousema, Fitsum G. Tadesse

**Affiliations:** 1 Armauer Hansen Research Institute, Addis Ababa, Ethiopia; 2 Radboud University Medical Centre, Nijmegen, The Netherlands; 3 London School of Hygiene and Tropical Medicine, London, United Kingdom; 4 Department of Medical Parasitology, School of Biomedical and Laboratory Sciences, University of Gondar, Gondar, Ethiopia; 5 Department of Biology, Arba Minch University, Arba Minch, Ethiopia; 6 Department of Microbial Sciences and Genetics, College of Natural and Computational Sciences, Addis Ababa University, Addis Ababa, Ethiopia; University of Florida, UNITED STATES OF AMERICA

## Abstract

In co-endemic regions, mixed *Plasmodium vivax (Pv)* and *Plasmodium falciparum (Pf)* infections are commonly reported. How mixed species infections compare to single species infections in terms of parasite densities and transmission to mosquitoes is poorly understood. Parasitemia, gametocytemia, and mosquito infectivity were evaluated among *Pv* mono-infections (n = 284), *Pf* mono-infections (n = 150), and mixed-*Pv-Pf* infections (n = 77) recruited at four Ethiopian health facilities. Parasitemia and gametocytemia were quantified in patient blood samples by qPCR. Mosquito infectivity was assessed using direct membrane feeding assays (DMFA), with *Plasmodium* species confirmation by quantitative PCR. *Pf* gametocyte prevalence was lower in mixed-infections (64.5%, 49/76) compared with mono-infections (91.8%, 135/147). Gametocyte density among positive carriers was also lower in mixed-infections (*p* < 0.001). *Pv* gametocyte prevalence was similar in mono- and mixed-infections despite lower asexual parasite density in mixed infections (*p* < 0.001). Statistically significant positive correlations between asexual parasitemia and gametocytemia were observed in mono-infections (*p* < 0.001), but not in mixed-species infections (*p* = 0.120 for *Pv*; and *p* = 0.570 for *Pf*). The overall proportion of infectious feeds to mosquitoes was high across infection types. Among mixed *Pv-Pf* parasite carriers, 56.3% (27/48) transmitted both species, often with both species being transmitted to individual mosquitoes. Species-specific gametocyte density remained associated with mosquito infection rates, and these associations were not altered by concurrent infection with the other species. Thus, mixed-species infections were associated with altered asexual and gametocyte densities, notably reduced gametocyte biomass, while mosquito infectivity was not reduced. Because mixed infections are often undetected, they represent a hidden risk for sustaining malaria transmission.

## Author summary

Malaria remains a major public health challenge in Ethiopia, where *Plasmodium vivax* and *Plasmodium falciparum* often occur together in the same patient. While mixed-species infections are common, little is known about how they compare to single-species infections in terms of parasite levels and transmission to mosquitoes. In this study, we examined parasite and gametocyte densities and transmissibility to mosquitoes of *P. vivax*, *P. falciparum*, or mixed infections across four health facilities. *P. falciparum* gametocyte prevalence and densities were lower in mixed infections than in single-species infections, whereas *P. vivax* gametocyte prevelance was similar across infection types despite lower densities in mixed infections. Importantly, patients with mixed infections remained highly infectious to mosquitoes, and many transmitted both species simultaneously. These findings show that mixed infections, which are often missed by routine diagnosis, can reduce parasite and gametocyte biomass while still contributing substantially to onward transmission. Recognizing and addressing mixed-species infections is therefore critical for malaria control and elimination efforts.

## Introduction

Malaria transmission in regions where *Plasmodium falciparum* and *Plasmodium vivax* are sympatric is characterized by a striking epidemiological paradox. *P. vivax* incidence and prevalence are often lower than *P. falciparum* [1]*,* despite *P. vivax* possessing several biological traits that theoretically favor persistence and spread. These biological traits include rapid gametocyte production within 2–3 days of blood-stage infection [2], efficient transmission at lower parasite densities [3], relapse-driven replenishment of the infectious reserviour [4], and accelerated sporogonic development across a wide range of temperature that enables it to complete sporogony within the lifespan of the vector. Since *P. vivax* possesses a dormant liver stage (hypnozoites) and individuals are often exposed to both *P. falciparum* and *P. vivax*, treatment of *P. falciparum* infections is frequently followed by subsequent *P. vivax* episodes.

As control interventions increasingly suppress *P. falciparum* transmission, *P. vivax* has emerged as important cause of malaria in many endemic regions [5]. Because both species are typically transmitted by the same *Anopheles* mosquitoes, understanding why *P. vivax* does not dominate under favourable conditions remains central to unravelling their shared epidemiology. One potential explanation for the above-described paradox may lie in interactions between co-circulating species or in challenges with accurate diagnosis. Routine surveillance substantially underestimates co-infections due to diagnostic limitations: microscopy detects only 5–20% of mixed *P. falciparum–P. vivax* infections, whereas molecular diagnostics reveal prevalences of up to 30% [6–12].

Contrasting with regular detection of co-infections in human parasite carriers, mixed species infections are rarely reported in wild-caught mosquitoes. This raises questions whether simultaneous transmission of both species to mosquitoes is inefficient and whether co-infection may affect the production of gametocytes or their infectivity. Interestingly, *P. malariae* co-infection is associated with increased *P. falciparum* gametocyte carriage [13] and vice-versa [14]. Whether *P. falciparum* and *P. vivax* interact in ways that modulate gametocyte development, competitive dynamics, or transmission potential remains unknown. In general, the transmission of non-falciparum parasites remains relatively understudied [15].

In this study, we examine whether mixed-species infections alter transmission potential by investigating asexual parasite and gametocyte densities among naturally acquired mixed *P. falciparum–P. vivax* infections and mono-infections and directly assessing transmission to *Anopheles* mosquitoes using mosquito feeding assays.

## Methods

### Ethical approval

Ethical approval was obtained from the AHRI/ALERT Ethics Review Committee (Ref. No. P042/18, and Ref. No. PO-058–24), the National Research Ethics Review Committee (Ref. No. MoSHE/RD/141/1097/19), and London School of Hygiene and Tropical Medicine (LSHTM) (Ref. No. 22518). Before sample collection, all participants and/or the parents/guardians of the children provided written informed consent.

### Study area and patients

**Fig 1:**
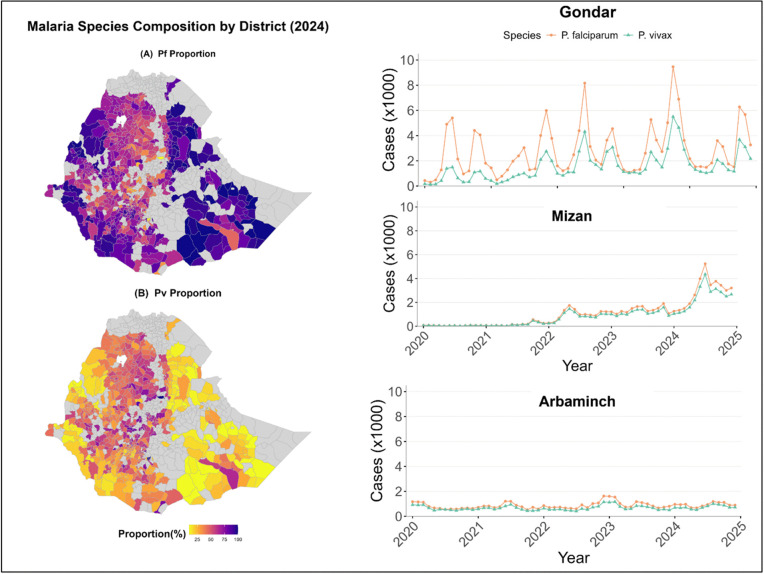
The map of the study sites and malaria case distribution over the past five years (2020–2025). **A)** shows the overall distribution of malaria cases throughout the country and the contribution of *P. falciparum* to the overall infection detected per district (proportion of *P. falciparum* cases divided by total malaria cases reported in each district) using data from the 2020–2025 Public Health Emergency Management (PHEM) system. Districts with no available data and malaria-free areas are shown in white, whereas increasing proportions of *P. vivax* infections are indicated using a color gradient. **B)** shows the overall distribution of malaria cases throughout the country and the contribution of *P. vivax* to the overall infection detected per district. Trend lines indicate the case flow over the past five years for the three selected sites. Direct link to the base layer: https://www.geoboundaries.org/countryDownloads.html License and terms of use information: https://www.geoBoundaries.org/index.html#license. The geoBoundaries dataset is openly available for research and publication use and is compatible with the journal’s CC BY 4.0 licensing requirements.

This study was conducted between 2022–2025 at four health centers in Ethiopia: Shele Health Center and Lante Health Centers in Arbaminch (southern Ethiopia), Maksegnit Health Center in Gondar (northwestern Ethiopia), and Mizan Health Center (southwestern Ethiopia) (Fig 1). Across these sites, self-presenting patients with signs and symptoms of malaria were screened, and those with microscopy-confirmed *P. vivax (Pv)* mono-infection, *P. falciparum (Pf)* mono-infection, or mixed*-Pv-Pf* infection were enrolled. Participants received treatment according to national guidelines. Patients infected by *P. vivax* were treated with chloroquine (total dose: of 25mg base/kg for 3 days) plus 14-day primaquine (0·25mg/kg body weight per day). For patients with *P. falciparum* mono-infection or mixed *Pv-Pf* infection*,* artemether-lumefantrine combined with a single low dose of primaquine was administered.

### Blood sample collection and parasite quantification

Prior to treatment, venous blood samples were collected into vacutainer EDTA tubes (using Precision-Glide Multi-sample Needles) for parasite and gametocyte quantification and Lithium heparin tubes for Direct Membrane Feeding Assay (DMFA). Blood samples in EDTA tubes were used to extract genomic DNA using Kingfisher Flex robotic extractor (Thermo Fisher Scientific). DNA was extracted from 50μL whole blood using MagMAX magnetic bead-based technology DNA multi-sample kit following the manufacturer’s protocol. Multiplex quantitative PCR (qPCR) targeting the 18S rRNA small subunit gene for *P. falciparum* and *P. vivax* was run using primer and probe sequences described before [16,17] using TaqMan Fast Advanced Master Mix (Applied Biosystems). *P. falciparum* parasites were quantified using standard curves generated from serial dilutions of NF54 ring stage parasites (10^6– 10^3 parasites/mL). For *P. vivax*, parasite quantification was done using recombinant plasmid constructs to infer copy numbers by running serial dilutions (10^7 – 10^3 copies/mL). Blood samples in protective buffer (RNA Protect Cell Reagent; Qiagen) were used to extract total RNA using MagMAX-96 Blood RNA Isolation Kit by MagMAX magnetic bead technology on a Kingfisher Flex robotic extractor (Thermo Fisher) following the manufacturer’s protocol. Reverse transcriptase quantitative PCR (RT-qPCR) targeting *P. vivax Pvs25* transcript and *P. falciparum* male (*PfMGET*) and female (*CCp4*) gametocyte mRNA transcripts were done using Luna Universal Probe One-Step RT-qPCR Kit (New England Biolabs, NEB) [18]. Gametocyte quantification for both species was achieved using *in vitro* RNA constructs in serial dilutions (10^8 – 10^3 copies/mL) [19,20]. Non-RT controls were included to monitor residual DNA amplification for *Pvs25*. The relative total *P. falciparum* gametocyte density was calculated by summing male and female gametocyte counts, which were quantified separately, with transcript counts converted to gametocyte numbers prior to summation (*PfMGET* for males, 9.8 transcripts per gametocyte, and *CCP4* for females, 4 transcripts per gametocyte) [18]. Four negative controls were included in each extraction batch in a 96 deep well format for both DNA and RNA extractions. Serial dilutions of the standard curves were generated in duplicate on each plate. The number of trendline concentrations differed between assays (4–6 orders of magnitude) but allowed estimation of species-specific parasite and gametocyte concentrations.

### Direct membrane feeding assay

Blood samples collected in Lithium Heparin tubes were used to feed colony maintained *Anopheles arabiensis* mosquitoes in DMFA following established procedures [21,22]. All materials used in DMFA were kept at 37°C before sample collection and feeding to mosquitoes. Briefly, blood in pre-warmed heparinized tubes was fed to 3–5 days old female mosquitoes that were starved for ~12 hours using mini-glass feeders (each 0.3mL capacity) that were covered with PARAFILM membrane (SIGMA-ALDRICH) and connected to a water bath at 39^o^C (Laan, Heiloo) for 25 minutes in the dark. Fully blood fed mosquitoes were maintained at 26-28^o^C and a relative humidity of 60–80% with 10% sugar solution, and were dissected for oocyst detection in the midgut that was stained in 1% mercurochrome on day 7 post feeding for *P. vivax,* and day 10 post feeding on *P. falciparum* and mixed species infected blood fed mosquitoes [23]. Approximately 30 mosquitoes were thus dissected for oocyst detection; additional mosquitoes from mixed species infected blood fed were kept alive until day 12 post-infection for further molecular analysis of sporozoites and processed if oocyst reading of d7-10 mosquitoes from the same experiment indicated successful transmission. This day was chosen to allow detection of late parasite development in mosquitoes (sporozoites), although we acknowledge that not all mosquitoes may have completed sporogonic development at this day. For sporozoite analysis on day 12, DNA was extracted following homogenization of entire mosquitoes by mini-bead beater based as previously described [24]. Briefly, whole mosquitoes were homogenized in 150 µl molecular-grade water with 0.2 g zirconium beads (1 mm diameter) and homogenized using a Mini-Bead Beater-96 (BioSpec). Part of the homogenate (50µL) was used for nucleic acid extraction using cetyl trimethyl ammonium bromide. Then, genomic DNA extracted from the whole mosquitoes was tested on a qPCR that targeted 18S small ribosomal subunit gene for parasite detection and quantification following the procedures explained above for blood samples.

### Data analysis

Statistical analyses were performed using STATA (version 17.0, Stata Corp., TX, USA) and R (version 4.5.1). Proportions were compared using one sample proportion test, and Pearson chi square test or Fisher exact test for independent observations. Equality tests with categorical variables were tested by two-sample Wilcoxon rank-sum (Mann-Whitney) test. Differences among independent groups were tested by Kruskal–Wallis test. Spearman rank correlation coefficient (ρ) was used to assess associations between continuous variables. Continuous variables were presented as medians and interquartile ranges (IQRs). The probability of mosquito infection was modeled as a function of gametocyte density using generalized additive mixed models (GAMMs) with a binomial distribution [20]. The response variable was specified as the number of infected mosquitoes out of the total number dissected. The model included log_10_-transformed gametocyte density and infection type (mono *vs* mixed) as fixed effects, and a random intercept for individual ID to account for repeated measures. A series of GAMMSs were fitted, with study site included as a fixed effect.

## Results

### Characteristics of study participants

A total of 511 individuals were enrolled for DMFA between 2022 and 2025 in Arbaminch, Gondar, and Mizan. Of these, 61.8% (316/511) were male, with a median age of 18 years (IQR: 12–25), and 43.1% (220/511) were febrile at recruitment. Microscopy showed substantial misclassification of mixed-species infections. Nearly half (48%; 37/77) of the mixed infections that were detected by PCR, were microscopically classified as mono infections. Consequently, PCR was used for the final classification and resulted in 284 (55.6%) of infections being *P. vivax* mono-infections, 150 (29.4%) *P. falciparum* mono-infections, and 77 (15.0%) mixed *Pv-Pf* infections. We observed strong positive correlation between microscopy and qPCR-estimated parasite densities for both *P. falciparum* and *P. vivax* while a weaker association was observed between *P. vivax* gametocyte density by microscopy and *Pvs25* transcripts copies by RT-qPCR (S1 Fig). No significant differences were observed in age or sex of individuals with mono- and mixed-species infections (Table 1).

**Table 1:** Characteristics of patients enrolled in the study.

Characteristics	*P. vivax* mono infection (N = 284)	*P. falciparum* mono infection (N = 150)	*P. vivax* and *P. falciparum* mixed (N = 77)
**Sex (male); % (n/N)**	60.2 (171/284)	65.3 (98/150)	61.0 (47/77)
**Age (median; IQR)**	15 (11 - 25)	19 (14-28)	17 (12-23)
**Under 5 years, % (n/N)**	0.7 (2/284)	3.3 (5/150)	2.6 (2/77)
**5 to 15 years, % (n/N)**	44.0 (125/284)	24.7 (37/150)	36.4 (28/77)
**15 years and above**	55.3 (157/284)	72.0 (108/150)	61.0 (47/77)
Microscopy agreement with PCR, % (n/N) *	96.5 (274/284)	98.7 (148/150)	52.0 (40/77)
**Fever at recruitment (axillary temperature ≥ 37.5** ^**o**^ **C), % (n/N)**	52.1 (148/284)	50.7 (38/75)	46.6 (34/73)

Abbreviations: IQR: Interquartile Range; NA: Not applicable; * The number of diagnoses accurately made by microscopy.

### Gametocyte densities are higher in mono-infections compared to mixed-species infections

**Fig 2:**
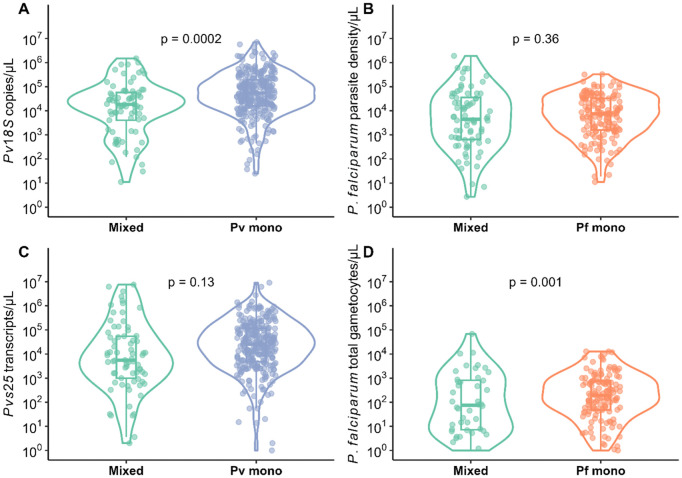
Parasite and gametocyte density comparison and association in the patients’ blood between mono and mixed infections for *P. vivax* and *P. falciparum.* **A)** Violin plot showing the median parasite density between *P. vivax* mixed (n = 48) and mono infections (284); **B)** Violin plot showing the median gametcoyte density between *P. vivax* mixed (n = 48) and mono infections (n = 276); **C)** Violin plot showing the median parasiste density between *P. falciparum* mixed (n = 48) and mono-infections (n = 150); **D)** Violin plot showing the median gametocytemia between *P. falciparum* mixed (n = 48) and mono-infections (n = 135).

**Fig 3:**
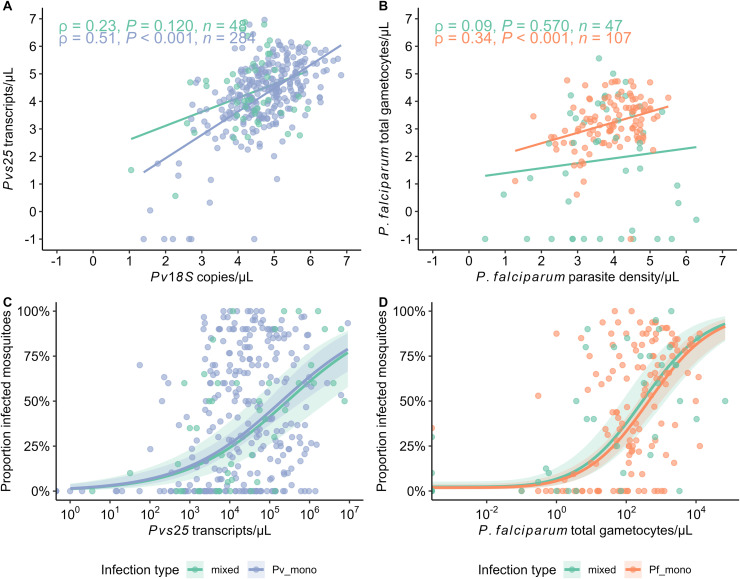
The Generalized Additive Mixed Models (GAMMs) for the prediction of mosquito’s infectivity from gametocytemia in both *P. vivax and P. falciparum* mixed- and mono-infections. **A)** Association between parasitemia and gametocytemia between *P. vivax* mono- and mixed-infections. The x-axis represents log₁₀ Pv18S copies/µL, and the y-axis represents Pvs25 transcripts/µL; **B)** Association between parasitemia and gametocytemia between *P. falciparum* mono- and mixed-infections. The x-axis represents log₁₀ parasites/µL, and the y-axis represents total gametocytes/µL, calculated as the sum of male and female gametocytes/µL; **C)** Proportion of infected mosquitoes (y-axis) against *P. vivax* gametocytemia (Log_10_ transformed Pvs25 transcripts/µL on x-axis); **D)** Proportion of infected mosquitoes (represented on y-axis) against *P. falciparum* total gametocytes/µL, calculated as the sum of male and female gametocytes represented on x-axis. For each infection, the number of infected mosquitoes out of the total dissected was used as the response variable. The model included log_10_-transformed gametocytemia and infection type (mono vs mixed infection) as fixed effects. A random intercept for individual infections was included to account for repeated measures.

Median *P. vivax* parasite density was higher in mono-infections (54,870 Pv18S copies/µL; Interquartile Range (IQR) 16,131 – 192,977) than in mixed-species infections (median 24,522 Pv18S copies/µL; IQR 9,267 – 66,291; *p* < 0.0002; Fig 2A). In contrast, *P. falciparum* parasite density did not differ significantly between mono infections (median 7,470 parasites/µL; IQR 1,589 – 33,307) and mixed-species infections (median 5,640 parasites/µL; IQR 755 – 33,356; *p* = 0.36; Fig 2C). *P. vivax* gametocyte positivity was high and comparable between *P. vivax* mono-infections (97.2%; 276/284) and mixed-species infections (93.5%; 72/77). However, for *P. falciparum*, gametocyte positivity was significantly higher in mono-infections (91.8%; 135/147) than in mixed-species infections (64.5%; 49/76) (*p* = 0.001) (Table 1). Similar to asexual parasite density, gametocyte density was higher in *P. vivax* mono-infections (median 24,160 *Pvs25* transcripts/µL; IQR 4,199 – 115,428) compared to mixed-species infections (median 11,110 *Pvs25* transcripts/µL; IQR 2,682 – 137,242) although this difference was not statistically significant (*p* = 0.13; Fig 2B). Gametocyte density was markedly higher in *P. falciparum* mono-infections (median, 140 gametocytes/µL; IQR 16 – 691) compared to mixed-species infections (median 49.5 gametocytes/µL; IQR 5 – 704; *p* < 0.001; Fig 2D). Parasite and gametocyte densities correlated positively in both *P. vivax* mono-infections (ρ = 0.40; *p* < 0.001) and *P. falciparum* mono-infections (ρ = 0.34; *p* < 0.001), but these associations were not apparent in mixed-species infections (Fig 3A and 3B).

### High infectivity to mosquitoes from *Plasmodium* mono- and mixed-species infections

Overall, 74.6% (381/511) of patients were infectious to mosquitoes (i.e., infecting ≥1 mosquito with ≥1 oocyst). The prevalence of infectious feeds was 75.4% (214/284) for *P. vivax* mono-infections, 72.0% (108/150) for *P. falciparum* mono-infections, and 76.6% (59/77) for *Pv–Pf* mixed infections. The median proportion of infected mosquitoes among infectious feeds was 56.7% (IQR: 26.7 – 83.3) for *P. vivax* mono, 64.8% (IQR: 34.4 – 81.3) for *P. falciparum* mono, and 63.3% (IQR: 30.0 – 78.1) for mixed species infections. Across all experiments, 40.8% (6,078/14,884) of mosquitoes became infected with a mean oocyst density of 13 (IQR: 5 – 37) in infected mosquitoes. The proportion of infected mosquitoes was not different between infection types *P. vivax* mono-infections (39%; 3192/8181) *vs P. falciparum* mono-infections (43.7%; 1978/4522) *vs Pv-Pf* mixed infections (41.6%; 908/2181). Similarly, mean oocyst density was not different (*p* = 0.5949); *P. vivax* mono infections (median: 11; IQR: 5 – 32) *vs P. falciparum* mono infections (median: 15; IQR: 5 – 54) *vs Pv-Pf* mixed infections (median: 15; IQR: 4 – 53); Tables 2 and S1. Oocyst count distributions are shown in S2 Fig and show a non-negligible number of infected mosquitoes with <5 oocysts.

**Table 2:** Mosquito infectivity across infection types and their gametocyte prevalences and densities at oocysts stage.

	*P. vivax* infected patients	*P. falciparum* infected patients	*P. vivax and P. falciparum* infected patients
***P. falciparum* gametocyte prevalence, % (n/N)**	NA	91.8 (135/147)*	64.5 (49/76)
***P. falciparum*, gametocytes/µL**	NA	140 (16–691)	4 (0–107)
***P. vivax* gametocyte prevalence, % (n/N)**	97.2 (276/284)	NA	93.5 (72/77)
***P. vivax,* Pvs25 transcripts/µL (median, IQR);**	24,160 (4,199–115,428)	NA	11,110 (2,682–137,242)
**Infectious feeds, % (n/N)**	75.4 (214/284)	72.0 (108/150)	76.6 (59/77)
**Infected mosquitoes, % (n/N)**	39 (3192/8181)	43.7 (1978/4522)	41.6 (908/2181)
Mean oocyst density**, (median (IQR)	11 (5- 32)	15 (5 - 54)	15 (4 - 53)

NA: Not Applicable.

*this shows that three samples from *P. falciparum* mono infections had no gametocyte data.

**this shows median of the mean oocyst density where mean oocyst density was first calculated for each feeding.

To further assess species-specific infectivity in mixed-species infections, 750 mosquitoes from 48 DMFAs using blood samples co-infected with mixed *Pv-Pf* infections were processed on day 12 for sporozoite detection, followed by 18S qPCR analysis of salivary glands. All of these 48 DMFAs resulted in mosquito infection at the oocyst level, as detected in mosquitoes from the same cage that were dissected on earlier days. Among these, 85.4% (41/48) of patients were confirmed to have transmitted parasites to at least one mosquito. The majority (65.9%; 27/41) transmitted both species, either concurrently to the same mosquito or to different mosquitoes, while the remainder transmitted only *P. vivax* (21.9%, 9/41) or only *P. falciparum* (12.2%, 5/41). The overall proportion of sporozoite-positive mosquitoes was 51.2% (384/750). Mosquito infection rates were highest in those carrying both parasite species (68.0%, 261/384) compared to those carrying only *P. falciparum* (15.9%, 61/384) or only *P. vivax* (16.1%, 62/384) (*p* < 0.001). *P. vivax* gametocytes were detected in all mixed-species infections (100%; 48/48). Individuals with mixed species infections who transmitted only *P. falciparum* had markedly lower *P. vivax* gametocytemia (median: 1,595 *Pvs25* transcripts/µL; IQR: 473 – 5,327) compared to those who transmitted only *P. vivax* (median: 48,974 *Pvs25* transcripts/µL; IQR: 15,000 – 96,807; *p* < 0.0001) or both species (median: 11,958 *Pvs25* transcripts/µL; IQR: 3,600 – 1,100,000; *p* < 0.001). *P. falciparum* gametocyte positivity (11.1%; 1/9) and density (70 gametocytes/µL in a single donor) were very low in those individuals who transmitted *P. vivax* only, while it was comparable between those only transmitting *P. falciparum* (median; 257 gametocytes/µL; IQR: 83 – 465) and those transmitting both species (median; 407 gametocytes/µL; IQR:7 – 1,765; *p* = 0.8493). Overall, non-infectious individuals exhibited lower gametocyte densities for both species (Table 3 and S3 Fig).

**Table 3:** Characteristics of mixed-species feeds with sporozoite outcomes.

Transmitted species	*P. vivax*	*P. falciparum*	*P. vivax and P. falciparum*	Non-infectious
**Infectious of feeds, % (n/N)**	18.8 (9/48)	10.4 (5/48)	56.3 (27/48)	14.6 (7/48)
**Infected mosquitoes, % (n/N)**	16.1 (62/384)	15.9 (61/384)	68.0 (261/384)	NA
**Sporozoite density, 18S qPCR, median (IQR)**	Pv: 314 (27–1,855) Pf: NA	Pv: NA Pf: 780 (26–6,884)	Pv: 78 (16–1,855) Pf: 484 (54–3,331)	NA
***Pv* gametocyte postivity, % (n/N)**	100 (9/9)	100 (5/5)	100 (27/27)	100 (7/7)
***Pf* gametocyte postivity, % (n/N)**	11.1 (1/9)	100 (5/5)	92.3 (25/27)	42.7 (3/7)
***Pv* parasite density, median (IQR)**	26,347 (7,203–64,680)	7,557 (391–9,267)	19,300 (9,336–67,901)	34,435 (28,545–237,315)
***Pv* gametocyte density, median (IQR)**	48,974 (15,000–96,807)	1,595 (473–5,327)	11,958 (3,600–1,100,000)	4,666 (653–10,284)
***Pf* parasite density, median (IQR)**	191 (40–32,168)	20,880 (1,3341–53,215)	4,822 (887–21,438)	16,090 (1,493–564,963)
***Pf* gametocyte density, median (IQR)**	70	257 (83–465)	407 (7–1765)	2; 4; 3,349**

NA: Not Applicable; *Shows percentage of individuals who infected at least one mosquito with ≥1 oocyst.

**Shows the list of gametocytemia for three infections that were positive of *P. falciparum* gametocytes*,* but were non-infectious to mosquitoes.

Finally, among individuals who transmitted both *P. vivax* and *P. falciparum,* most had high gametocyte densities for both species, facilitating dual transmission. Notably, 96.3% (26/27) of these patients with dual transmission, transmitted both parasites to single mosquitoes, although some mosquitoes carried only one of the two species. Sporozoite density, as determined by 18S qPCR, did not differ between those who infected mosquitoes with *P. vivax* mono- and mixed-species infections. Similarly, sporozoite density was comparable between mosquitoes infected with *P. falciparum* mono-infections and mixed-species infections (S4 Fig).

### Gametocyte density is the most important determinant of transmissibility to mosquitoes

The probability of mosquito infection was positively associated with gametocytemia for both *P. falciparum* and *P. vivax*, in both mono- and mixed-species infections. Using generalized additive mixed models (GAMMs), we predicted mosquito infection rates based on gametocyte density. For *P. falciparum*, each log₁₀ increase in gametocytemia was associated with a nearly threefold increase in the odds of mosquito infection (OR=2.96; 95% CI: 2.36 – 3.71; *p* < 0.001), with no significant difference observed between mono- and mixed-species infections (OR=0.82; 95% CI: 0.40 – 1.69; *p* = 0.596). Similarly, *P. vivax* gametocytemia was positively associated with proportion of infected mosquito (OR=2.26; 95% CI: 1.74 – 2.93; *p* < 0.001; for each log increase), again with no significant difference between mono- and mixed-infections (OR=1.11; 95% CI: 0.58 – 2.15; *p* = 0.774) (Fig 3C and 3D and S2 Table). After adjusting for parasite density, gametocyte density, age, and study site, mosquito infectivity did not differ significantly between mono- and mixed-species infections for either *P. vivax* (AOR = 0.65, 95% CI: 0.29 - 1.48; *p* = 0.307) or *P. falciparum* (AOR = 2.03, 95% CI: 0.93 - 4.42; *p* = 0.076) (S3 Table).

## Discussion

This study provides direct evidence on the relative infectiousness to mosquitoes of natural mixed species *Plasmodium* infections in co-endemic settings. *P. falciparum* gametocyte prevalence and density were lower in mixed infections than in *P. falciparum* mono-infections. *P. vivax* gametocyte prevalence was similarly high in mono- and mixed-infections, while asexual parasite and gametocyte densities were lower in mixed-infections. Mosquito infection rates were high across infection types and were explained by hight (species-specific) gametocyte estimates, with no detectable reduction in mosquito infectivity attributable to co-infection after accounting for gametocyte measures.

Parasite densities differ markedly between *P. falciparum* and *P. vivax* infections. *P. vivax* parasitemia is generally low due to its strict preferences for reticulocytes that represent only 1–2% of circulating erythrocytes [25,26]. This limited niche makes *P. vivax* highly vulnerable to factors that reduce reticulocyte availability. *P. falciparum* might exacerbate this constraint through its association with anemia [27] and dyserythropoiesis [28], potentially restricting reticulocyte availability. By comparison, *P. falciparum* has a competitive advantage due to its broader invasion range (young and mature erythrocytes) and higher multiplication rate (16–32 merozoites per schizont *vs* 12–24 for *P. vivax*) [29,30]. Together, these mechanisms may explain why *P. vivax* parasite density is low, especially in mixed-species infections. An important observation from our study is that microscopy missed approximately half of the mixed infections that were detected by qPCR. This diagnostic gap has significant implications for malaria case management in co-endemic settings like Ethiopia, where *P. falciparum* and *P. vivax* require distinct treatment regimens: chloroquine plus a 14-day course of primaquine for radical cure of *P. vivax*, versus artemether-lumefantrine plus a single low dose of primaquine for *P. falciparum*. Failure to detect mixed infections therefore risks inappropriate treatment, incomplete radical cure, and continued transmission. National malaria control programs in co-endemic regions should therefore consider strengethening diagnostic capacity to ensure accurate species identification or optimize treatment startegies such as a unified treatment for the two species in co-endemic settings as some countries are already doing it [31,32].

Despite differences in parasite density, *P. vivax* gametocyte prevalence was similarly high in mixed and mono-infections, indicating efficient gametocyte production regardless of parasitemia [33]. *P. vivax* gametocyte density broadly followed asexual parasite densities and was lower in mixed-species infections. In contrast, *P. falciparum* showed lower gametocyte positivity and density in mixed infections, consistent with prior observations that co-infection may suppress *P. falciparum* gametocyte carriage [14,34].

Uniquely, our study combined quantitative measures of parasite and gametocyte signals in human blood with DMFAs to assess onward transmission to *An. Arabiensis* mosquitoes. For both species, the probability that a feed produced infected mosquitoes increased sharply with gametocyte density [35]. Mixed-species infection donors frequently transmitted both *P. vivax* and *P. falciparum*, generally explained by species-specific gametocyte densities, often both species transmitted to the same mosquito. Thus, under the conditions of our assays, the vector readily acquired and supported both parasites from a single bloodmeal, and we found no clear evidence that co-infection reduced mosquito infection rates in a manner that was independent of gametocyte density. This observation is consistent with prior reports of concurrent transmission from mixed-species blood samples [36] and argues against competition between species inside the mosquito in our experimental system. Lower prevalence of *P. vivax* observed in wild-caught mosquitoes [37] and lower incidence of *P. vivax* in some co-endemic populations are therefore unlikely to be (fully) explained by vector-level competition. Instead, *P. falciparum*-induced anemia, dyserythropoiesis, immune-mediated effects, or competition for reticulocytes could reduce *P. vivax* asexual expansion, and thereby lower gametocyte biomass availability for uptake by mosquitoes [38–40]. These host-level processes, although not directly measured here, provide a coherent explanation for the reduced *P. vivax* parasite and gametocyte densities in mixed infections while mosquito receptivity to both species remained high.

Similar mosquito infection prevalence in mono and mixed infections despite lower gametocyte specific transcripts in mixed infections may reflect (i) the non-linear relationship between gametocyte biomass and mosquito infection (low densities can still infect and increases show diminishing returns), (ii) variation in transcript copies per gametocyte and sex specific composition (male:female ratios and differing transcript copy numbers per gametocyte can alter infectivity per unit female marker), or (iii) inter-species interactions or host immune modulation that alter gametocyte maturation or activation.

Although we report that dual transmission occurs, including both parasites being detected within single mosquitoes, mixed-infections remain rare in wild-caught *Anopheles* mosquitoes [41,42]. This scarcity likely reflects multiple factors: asynchronous gametocyte production between species or diagnostic under-detection of minority infections [11]. If *P. vivax* infections would typically be of shorter duration than *P. falciparum* infections, this could explain why mosquitoes are rarely detected with both sporozoite species. However, the sparse studies that examined infection duration in co-endemic settings suggest that *P. vivax* infections may in fact last longer than *P. falciparum* [43].

Our analysis used naturally acquired infections and single time-point mosquito feeding assays, which provide biologically relevant human-to-mosquito infectivity estimates but preclude inference about temporal dynamics (e.g., gametocyte conversion, maturation, parasite multiplication rates, or infection duration). Gametocyte and parasite quantification for *P. vivax* were transcript-based (*Pvs25*) and recombinant plasmid-derived, so values represent relative abundance rather than absolute cell counts and cannot be directly compared to *P. falciparum*. For *P. falciparum* we applied sex-specific transcript assays with available calibration approaches, but validated transcript to cell conversion factors for *P. vivax* are lacking. Additionally, we did not measure the actual number of parasites or gametocytes ingested by mosquitoes, previous studies demonstrated strong correlations between gametocyte concentrations of *P. falciparum* measured in the blood and the mosquito blood meal immediately after engorging [44]. We quantified sporozoite stage parasites using the whole mosquitoes collected at day 12 post-infection. This approach introduces uncertainty, as the signal may derive from both salivary gland and oocyst sporozoites. Given that *P. falciparum* sporogony can extend beyond day 12 while *P. vivax* develops more rapidly, this limitation should be considered when interpreting our results. Finally, mixed infections were less frequent than mono-infections, limiting power for some subgroup analyses. Our experiments thereby do not answer all questions on species interactions. Together, these constraints mean our results describe prevalence–density patterns and their association with mosquito infection at a single time point but do not establish the causal within-host mechanisms; resolving those questions would benefit from longitudinal studies that ideally capture incident infections and the effect of co-infection on gametocyte dynamics and infectivity.

Taken together, our results indicate that mixed infections can reduce parasite and gametocyte biomass in the human host but do not necessarily reduce the ability of mosquitoes to become infected from those hosts.

## Supporting information

S1 Fig Correlation between microscope results and qPCR/qRT-PCR results for parasite density (asexual stages) and gametocyte (sexual stage) densities in mono infections of *P. falciparum* and *P. vivax.* Scatter plots illustrate the relationship between parasite densities quantified by molecular methods (qPCR or RT-qPCR) and those observed by microscopy. Each panel shows log10-transformed values with regression lines (red) (lm fit) and 95% confidence intervals (gray); **A**) shows *P. falciparum* asexual parasite density measured by microscopy compared with qPCR; **B**) shows *P. falciparum* gametocyte density measured by microscopy compared with RT-qPCR; **C)** presents *P. vivax* asexual parasite density measured by microscopy compared with qPCR (18S copies/µL); **D)** illustrates *P. vivax* gametocyte density measured by RT-qPCR (*Pvs25* transcripts copies/µL) compared with microscopy.
(TIF)

S2 Fig Distribution of mean oocyst density by infection type for infectious individuals. **A**) Histogram showing the distribution of mean oocyst density across mixed, *Pf mono*, and *Pv mono* infections for all counts (range 0–300); **B**) Histogram showing the distribution of mean oocyst density restricted to values between 0 and 20, comparing mixed, *Pf mono*, and *Pv mono* infections to show clearly frequency of individuals with fewer oocysts per midgut.
(PNG)

S3 Fig *P. falciparum* and *P. vivax* gametocyte densities for mixed species feeds. *P. vivax* (blue) and *P. falciparum* (orange) gametocyte densities for mixed *P. vivax* and *P. falciparum* infections offered to mosquitoes in DMFAs are indicated in box plots. The whisker plot indicates the distance between the Interquartile range values in the dataset with the middle line indicating the median value of the gametocyte density.
(TIF)

S4 Fig Comparison of the parasite density in mosquitoes at sporozoite stage. It was determined by 18sqPCR between mono and mixed species infected mosquitoes for *P. vivax* and *P. falciparum*.
(TIF)

S1 Table Mosquito infectivity across infection types and their gametocyte prevalences and densities stratified by sites. (DOCX)

S2 Table The Odds Ratio of mosquitoes’ infection rate based on gametocyte density for both *P. vivax* and *P. falciparum* mixed infection and mono infections. (DOCX)

S3 Table Adjusted models assessing factors associated with mosquito infectivity in *P. vivax* and *P. falciparum.* (DOCX)
