# Supplementary material for: Plasmodium vivax and Plasmodium falciparum mixed infections in human and mosquito hosts: The impact of multi-species infection on parasite densities and transmission to mosquitoes
Source: PLoS Negl Trop Dis. 2026 Jul 24;20(7):e0014148. doi: 10.1371/journal.pntd.0014148 (PMC13446731; doi:10.1371/journal.pntd.0014148)
Supplement: Supplementary file 1 [file pntd.0014148.s005.docx]

**S1 Table. The Odds Ratio of mosquitoes’ infection rate based on gametocyte density for both *P. vivax* and *P. falciparum* mixed infection and mono infections.**

| Variables | OR (95% CI) | *p-*value |
| --- | --- | --- |
| *P. falciparum* infectivity |  |  |
| Gametocyte density increase (log_10_ scale) | 2.96 (2.36–3.71) | <0.001 |
| Reference (*P. falciparum* mixed infection) |  |  |
| *P. falciparum* mono infections | 0.82 (0.40–1.69) | 0.596 |
| *P. vivax* infectivity |  |  |
| Gametocyte density increase (PvS25 copies/µL) (log_10_ scale) | 2.26 (1.74–2.93) | <0.001 |
| Reference (*P. vivax* mixed infections) |  |  |
| *P. vivax* mono infections | 1.11 (0.58–2.15) | 0.774 |
