# Supplementary material for: Plasmodium vivax and Plasmodium falciparum mixed infections in human and mosquito hosts: The impact of multi-species infection on parasite densities and transmission to mosquitoes
Source: PLoS Negl Trop Dis. 2026 Jul 24;20(7):e0014148. doi: 10.1371/journal.pntd.0014148 (PMC13446731; doi:10.1371/journal.pntd.0014148)
Supplement: Supplementary file 2 [file pntd.0014148.s006.docx]

**S3 Table. Adjusted models assessing factors associated with mosquito infectivity in *p. vivax* and *P. falciparum.***

|  | | *P. vivax* | | *P. falciparum* | |
| --- | --- | --- | --- | --- | --- |
| Model | Covariate | OR (95% CI) | P-value | OR (95% CI) | P-value |
| Model 1 | Gametocyte density | 2.30 (1.79–2.97) | <0.001 | 2.79 (2.22–3.50) | <0.001 |
|  | Parasite density | 0.90 (0.65–1.25) | 0.522 | 1.36 (0.98–1.89) | 0.070 |
| Model 2 | Gametocyte density | 2.43 (1.94–3.03) | <0.001 | 3.01 (2.38–3.79) | <0.001 |
|  | Age: Above 15 years | 4.89 (3.01–7.95) | <0.001 | 1.20 (0.64–2.24) | 0.572 |
|  | **Mixed infection** | 0.82 (0.41–1.65) | **0.581** | 1.26 (0.60–2.62) | **0.541** |
| Model 3 | Gametocyte density | 2.21 (1.78–2.76) | <0.001 | 2.96 (2.36–3.71) | <0.001 |
|  | **Mixed infection** | 0.89 (0.43–1.86) | **0.762** | 1.21 (0.59–2.50) | **0.597** |
| Model 4 | Gametocyte density | 2.38 (1.85–3.05) | <0.001 | 2.87 (2.27–3.62) | <0.001 |
|  | Parasite density | 1.06 (0.77–1.48) | 0.710 | 1.41 (1.00–1.98) | 0.050 |
|  | Age: Above 15 years | 4.98 (3.03–8.17) | <0.001 | 1.38 (0.73–2.62) | 0.322 |
|  | **Mixed infection** | 0.84 (0.41–1.73) | **0.643** | 1.26 (0.61–2.62) | **0.531** |
| Model 5 | Gametocyte density | 2.12 (1.66–2.70) | <0.001 | 2.36 (1.78–3.14) | <0.001 |
|  | Parasite density | 1.31 (0.95–1.81) | 0.101 | 1.53 (1.08–2.17) | 0.017 |
|  | Age: Above 15 years | 3.09 (1.91–4.99) | <0.001 | 1.25 (0.66–2.35) | 0.490 |
|  | Site: Gonder | 2.71 (1.01–7.26) | 0.047 | 12.80 (2.69–60.99) | 0.001 |
|  | Site: Mizan | 10.39 (5.50–19.64) | <0.001 | 15.00 (2.76–81.42) | 0.002 |
|  | **Mixed infection** | 0.65 (0.29–1.48) | **0.307** | 2.03 (0.93–4.42) | **0.076** |

***OR odds ratio; CI, confidence interval. Model 5 represents the fully adjusted model including gametocyte density, parasite density, age category, infection type, and study site (Arba Minch used as reference), with participant ID included as a random effect.***
