# Supplementary material for: Plasmodium vivax and Plasmodium falciparum mixed infections in human and mosquito hosts: The impact of multi-species infection on parasite densities and transmission to mosquitoes
Source: PLoS Negl Trop Dis. 2026 Jul 24;20(7):e0014148. doi: 10.1371/journal.pntd.0014148 (PMC13446731; doi:10.1371/journal.pntd.0014148)
Supplement: Supplementary file 3 [file pntd.0014148.s007.docx]

**S1 Table. Mosquito infectivity across infection types and their gametocyte prevalences and densities** **stratified by sites**

|  | ***P. vivax* infected patients (N=284)** | | | ***P. falciparum* infected patients (N=150** | | | ***P. vivax and P. falciparum* infected patients (N=77)** | | |
| --- | --- | --- | --- | --- | --- | --- | --- | --- | --- |
| **Study sites** | **Arba Minch** | **Gonder** | **Mizan** | **Arba Minch** | **Gonder** | **Mizan** | **Arba Minch** | **Gonder** | **Mizan** |
| **Total participants (%, n/N)** | 79.6  (226/284) | 2.8  (8/284) | 17.6 (50/284) | 3.3  (5/150) | 87.3 (131/150) | 9.3 (14/150) | 36.4  (28/77) | 41.5 (32/77) | 22.1 (17/77) |
| ***P. falciparum* gametocyte prevalence, % (n/N)** | NA | NA | NA | 60.0  (3/5) | 96.9 (127/131) | 35.7  (5/14) | 42.9  (12/28) | 90.6 (29/32) | 47.1 (8/17) |
| ***P. falciparum*, gametocytes/µL** | NA | NA | NA | 1.1  (0 - 16) | 191.5  (47 - 733) | 0  (0 - 5.1) | 0  (0 - 1.7) | 465  (15 - 1953) | 0  (0 - 7.4) |
| ***P. vivax* gametocyte prevalence, % (n/N)** | 96.5  (218/226) | 100  (8/8) | 100  (50/50) | NA | NA | NA | 96.4  (27/28) | 93.8 (30/32) | 88.2 (15/17) |
| ***P. vivax,* Pvs25 transcripts/µL (median, IQR);** | 22,999  (3286–122,503) | 830,316  (399,401 - 351, 401) | 19,426  (5670 - 60,523) | NA | NA | NA | 7939  (679 - 23,645) | 5164  (1242 - 332,903) | 4647  (66 - 10,821) |
| **Infectious feeds % (n/N)** | 71.2  (161/226) | 87.5  (7/8) | 92.0  (46/50) | 0  (0/5) | 77.9 (102/131) | 4.9  (6/14) | 64.3  (18/28) | 81.3 (26/32) | 88.2 (15/17) |
| **Infected mosquitoes, % (n/N)** | 32.0 (2177/6801) | 58.8  (141/240) | 76.7 (874/1140) | 0  (0/100) | 46.0 (1909/4152) | 25.6 (69/270) | 20.7 (173/835) | 55.3 (545/986) | 52.8 (190/360) |
| **Mean oocyst density, median (IQR)** | 4  (0- 14) | 50  (22-85) | 21  (7 -34) | 0 | 8  (2-42) | 0  (0-6) | 2  (0-14) | 24  (3 - 85) | 7  (2 -23) |
