# Supplementary material for: Plasmodium vivax and Plasmodium falciparum mixed infections in human and mosquito hosts: The impact of multi-species infection on parasite densities and transmission to mosquitoes
Source: PLoS Negl Trop Dis. 2026 Jul 24;20(7):e0014148. doi: 10.1371/journal.pntd.0014148 (PMC13446731; doi:10.1371/journal.pntd.0014148)
Supplement: Supplementary file 4 [file pntd.0014148.s008.docx]

**Review of Chali et al.: “*Plasmodium vivax* and *Plasmodium falciparum* mixed infections in human and mosquito hosts: the impact of multi-species infection on parasite densities and transmission to mosquitoes”.**

This study examined the impact of Plasmodium vivax (Pv) and Plasmodium falciparum (Pf) mixed-species infections on parasite densities, gametocytemia, and transmission to mosquitoes in Ethiopian field settings. The study is well-designed and addresses an important and underexplored question in malaria epidemiology. Using a large cohort (n=511) combined with molecular quantification and direct membrane feeding assays (DMFAs), the authors found that the gametocyte levels of Pf in mixed Pf-Pv infections are lower than mono-Pf whereas mono-Pv had similar gametocyte level with mixed Pf-Pv infections. Both mixed- and mono-infections showed similar infectivity and contribute equally to transmission.

The integration of molecular diagnostics with functional transmission assays is a major strength and provides valuable data on how mixed infections influence human-to-mosquito transmission dynamics. However, some of the analyses and interpretations require clarification and strengthening. In particular, the conclusion that there is “no competition” between species is overstated, and the statistical analyses do not fully account for potential confounders or interaction effects. Addressing these issues will substantially improve the rigor and impact of the manuscript. Below are my comments and suggestions:

**Abstract**

L26: Replace the phrase “…incompletely understood” to “… poorly understood”.

L36-37: The sentence ‘…Transmission to mosquitoes was high across infections and infection combinations…” needs to be clearer. What is the reference to say high as 56.3% infection rate in mosquitoes is not that high?

**M&M**

L148-151: It is unclear how many mosquitoes per patient blood were fed on, given the mosquito infection prevalence might vary by mosquito numbers. How many mosquitoes remain post-day10 dissection?

L152: Concern if 12 instead of 14 days post infection was sufficient for complete sporozoite development from oocysts inside the mosquito. This will affect sporozoite count in salivary gland.

L167-171: Concern that key covariates such as study site heterogeneity, age of patients, parasite density are not addressed or adjusted in the analyses when comparing infectivity.

**Results**

L175: The percentage is not equal with the number in parenthesis, 50.9% (220/511), please check and correct it.

L178: Suggest present prevalence and infectivity data stratify be study sites as suppl data.

L187-202: The total parasite and gametocyte densities were compared between mono and mixed *Pf* and *Pv* infections. But it is unclear how parasite/gametocyte densities were measured? By microscopy or qPCR? If by qPCR, need to describe the calculation of how to transform CT values into parasite or gametocyte/ul of blood using standard serial dilution and whether absolute or relative quantification was considered in the Method.

L198-200: Check median and p-value as 2D seems to show overlapping median and range like that in 2B.

L210-216: In Fig 3, why the unit are different e.g., panel A presents transcript copies/ul while panel B present gametocytes/ul? Likewise, inconsistence between 3A & B regarding Pv18s copies vs. parasite density.

Also, in panel B, it is not clear whether the parasitemia/gametocytema represents both species or *Pf* only. The statistical values in the figure are not readable and some gene names like *Pvs25* needs to be italicized.

L218: how to calculate the sum of male & female gametocytes.

L227: “*falciparum* mono-infections **this** was 72.0% (108/150)…” remove “this” or rephrase the statement.

L225-228: Unclear how many patients were included in DMFA? all 511 or only gametocyte-positive patients? Was 14,884 the total mosquitoes being fed in DMFA? If so, it's ~30 mosquitoes per patient for all 511 patients in each feed, which is way too low given 30 were used for day7/10 oocyst dissection and remaining for day12 sporozoite assessment.

L230: Unclear how many patient samples this derived from, all 284 mono-Pv? as there could be inter-patient variation in mosquito infectivity, would be important to show the range of the proportion of infected mosquito for each infection type.

L247-248: This is unclear. Should this be rewrite as among the 384 sporozoite-positive mosquitoes, how many were detected with Pv+Pf, Pv only, and Pf only. Also, should the denominator the same (384 or 386 mosquitoes)? Why the denominator for both species is 386, Pf is 90, and Pv is 134?

Table 3: Again, why the denominator is different? should this be based on total # of mosquitoes fed on the 48 mixed Pv-Pf patients, and among the total, how many were Pv-sporozoite infected only, Pf-sporozoite infected only, and Pv/Pf-sporozoite infected.

**Discussion**

L298: Typo “…may explain why especially**”**.

L300-306: It seems a conflicting idea. While the prevalence of *Pv* gametocyte is similar in mixed and mono infections, it is an overstatement that gametocyte production is efficient. This could just mean almost all *Pv* infections had gametocytes (typical asynchronous and early development features of *Pv*). On the other hand, *Pv* and *Pf* gametocyte densities were lower in mixed infections. The lower gametocyte densities seem to contradict the notion of efficient gametocyte production.

L42, 315-323: The conclusion of “no competition” between *Plasmodium* species could be overinterpreted. *Pf* gametocyte prevalence and density are **reduced in mixed infections and** *Pv* asexual density is also **reduced in mixed infections.** This suggests perhaps some **competition (or interaction) in the human host**, even though gametocyte infectivity is similar in the mosquitoes.
