# Supplementary material for: Plasmodium vivax and Plasmodium falciparum mixed infections in human and mosquito hosts: The impact of multi-species infection on parasite densities and transmission to mosquitoes
Source: PLoS Negl Trop Dis. 2026 Jul 24;20(7):e0014148. doi: 10.1371/journal.pntd.0014148 (PMC13446731; doi:10.1371/journal.pntd.0014148)
Supplement: Supplementary file 5 [file pntd.0014148.s010.docx]

**Response to Reviewers**

**Journal Requirements:**

1) We do not publish any copyright or trademark symbols that usually accompany proprietary names, eg ©, ®, or TM (e.g. next to drug or reagent names). Therefore, please remove all instances of trademark/copyright symbols throughout the text, including: - ® on page: 8.

**Response #1:** Thank you for your comments. We now removed all copyright or trademark symbols that were previously used in the manuscript (Line 123 – 153).

2) We notice that your supplementary Tables are included in the manuscript file. Please remove them and upload them with the file type 'Supporting Information'. Please ensure that each Supporting Information file has a legend listed in the manuscript after the references list.

**Response #2:** We now removed the table to the file “Supporting information” and included a legend in the manuscript at the recommended place.

3) Figure 1: please (a) provide a direct link to the base layer of the map (i.e., the country or region border shape) and ensure this is also included in the figure legend; and (b) provide a link to the terms of use / license information for the base layer image or shapefile. We cannot publish proprietary or copyrighted maps (e.g. Google Maps, Mapquest) and the terms of use for your map base layer must be compatible with our CC BY 4.0 license.

Note: if you created the map in a software program like R or ArcGIS, please locate and indicate the source of the basemap shapefile onto which data has been plotted. If your map was obtained from a copyrighted source, please amend the figure so that the base map used is from an openly available source. Alternatively, please provide explicit written permission from the copyright holder granting you the right to publish the material under our CC BY 4.0 license. If you are unsure whether you can use a map or not, please do reach out and we will be able to help you. The following websites are good examples of where you can source open access or public domain maps: * U.S. Geological Survey (USGS) - All maps are in the public domain. (<http://www.usgs.gov>)
* PlaniGlobe - All maps are published under a Creative Commons license so please cite “PlaniGlobe, <http://www.planiglobe.com>) CC BY 2.0” in the image credit after the caption. (<http://www.planiglobe.com/?lang=enl>) * Natural Earth - All maps are public domain. (<http://www.naturalearthdata.com/about/terms-of-use/>).

**Response #3**: We provided these details in the revised manuscript. The Ethiopia administrative boundary layer (ADM3) used as the base map in Figure 1 was obtained from the geo-boundaries open-access database. Direct link to the base layer: <https://www.geoboundaries.org/countryDownloads.html>. License and terms of use information: <https://www.geoBoundaries.org/index.html#license>. The geoBoundaries dataset is openly available for research and publication use and is compatible with the journal’s CC BY 4.0 licensing requirements. The source and licensing information have now been included in the revised figure legend; lines 108 – 119.

**Reviewers' Comments:**

**Reviewer #1:** Minor comments methodology:

1. Molecular quantification of parasites is a main outcome variable in the study for both gametocytes and asexual parasites. Undoubtedly, this is done at a sufficient quality here. Still, reconstitution of standard curves for asexual parasites should span at least 6 magnitudes (eg 10^8-10^3 as done for the gametocytes), with identical dilution series for Pf and Pv. This would improve the quality of parasite quantification. It is logic, that for P. vivax plasmids are used. However, if the relation between copy numbers and parasite densities taking the copy number of the 18s gene in *P. vivax* is not taken into consideration, this is not really a parasite quantification rather an estimation based on a quantification of copy numbers. This should be mentioned in the methods section. One could think of mention this as a limitation of the study in the discussion.

**Response #4:** We appreciate the thorough review of reporting of the parasite quantification methodology. We agree that trendlines vary in the number of magnitudes included. Our decision to use the five magnitudes (10^7 – 10^3) for *P. vivax* (plasmid standards) and four magnitudes (10^6 – 10^3) for *P. falciparum* (NF54 cultured *P. falciparum* asexual stages) were informed by our prior studies to span the densities that are commonly observed in natural infections in our setting (Tadesse *et al.*, 2026; Tadesse *et al.*, 2018). For gametocytes, our choice of six magnitudes (10^8 – 10^3) is in line with the recommended approach here. The calculation is obviously based on the slope of the curve for quantification of samples; we provide the individual results derived from a total of ten experiments in the table below. For each experiment, the standards were run in duplicate for each stage of the dilution series. It is evident from the below table that the results were consistent between experiments (Standard deviation ranging from 0.30 to 0.75). To account for the between experiments variations, we used the average CT values for each serial dilution to generate the standard curve that was finally used to calculate copy numbers for *P. vivax* and parasite density for *P. falciparum.*

**Table: average CT values of duplicate experiments run on each 96 well plate format (Pv18S)**

| **Dilution** | **01** | **02** | **03** | **04** | **05** | **06** | **07** | **08** | **09** | **10** | **STDEV** | **Mean** |
| --- | --- | --- | --- | --- | --- | --- | --- | --- | --- | --- | --- | --- |
| 10^7 | 26.3 | 26.4 | 26.5 | 26.1 | 26.5 | 25.9 | 25.6 | 26.4 | 26.3 | 26.5 | 0.30 | 26.3 |
| 10^6 | 30.2 | 30.5 | 31.1 | 29.1 | 29.2 | 29.5 | 29.5 | 29.6 | 29.6 | 30.0 | 0.62 | 29.8 |
| 10^5 | 33.5 | 33.8 | 33.7 | 32.7 | 32.8 | 32.9 | 34.0 | 32.9 | 32.9 | 33.3 | 0.47 | 33.3 |
| 10^4 | 36.9 | 37.3 | 37.6 | 36.7 | 36.3 | 36.3 | 36.4 | 37.2 | 36.6 | 36.3 | 0.47 | 36.8 |
| 10^3 | 39.9 | 39.2 | 39.5 | 39.7 | 38.1 | 39.5 | 40.1 | 39.9 | 38.1 | 40.1 | 0.75 | 39.4 |

**Table: average CT values of duplicate experiments run on each 96 well plate format (Pf18S)**

| 10^6 | 26.7 | 26.3 | 26.6 | 25.7 | 26.0 | 26.2 | 26.7 | 27.4 | 26.9 | 25.3 | 0.61 | 26.4 |
| --- | --- | --- | --- | --- | --- | --- | --- | --- | --- | --- | --- | --- |
| 10^5 | 30.1 | 29.9 | 30.5 | 29.2 | 29.3 | 29.4 | 30.3 | 30.4 | 30.3 | 29.1 | 0.55 | 29.9 |
| 10^4 | 33.5 | 33.4 | 34.1 | 32.5 | 32.5 | 32.7 | 33.5 | 33.5 | 33.9 | 32.6 | 0.60 | 33.2 |
| 10^3 | 37.0 | 36.7 | 37.2 | 36.2 | 36.0 | 35.9 | 36.4 | 38.0 | 36.9 | 36.1 | 0.66 | 36.6 |

The serial dilutions are per milliliter in both cases. Columns indicate the average CT values of 10 experiments followed by the standard deviation (STDEV) and the mean of all experiments.

The final standard curves used for the quantification were demonstrated below

Pv18S (recombinant plasmid)

|  | Pv18s 7 |
| --- | --- |
| 7 | 26.25 |
| 6 | 29.83 |
| 5 | 33.25 |
| 4 | 36.76 |
| 3 | 39.41 |

Pf18S (NF54 culture material)

|  | Pf18s |
| --- | --- |
| 6 | 26.38 |
| 5 | 29.85 |
| 4 | 33.22 |
| 3 | 36.64 |

Regarding *P. vivax*, we acknowledge that plasmid-based quantification does not directly equate to parasite density unless the copy number of the 18S gene is explicitly considered. Since we used the same approach across infection types, we assumed that no difference would be introduced with the same method of estimation and we have purposefully refrained from any estimates of parasite density. We acknowledge the reviewer’s comment that we can’t compare directly between the two species (*P. vivax* and *P. falciparum*) in terms of parasite density taking this into consideration. This has been clarified in the discussion section of the revised manuscript (lines 382 - 388). We also clarified our reasoning for the number of dilutions for each trendline in the Methods section.

2. Taking the importance of correct molecular quantification for the present study, RNA extraction should be well supported by citations, or the manufacturers protocol should be mentioned at least. Most importantly, the procedure for removal of residual DNA should be explained to assure that you are really quantifying gametocytes since pvs25 is also detectable as DNA not only RNA transcripts. It is recommendable to mention not only the use of negative controls (as you do correctly) but also the use of Non-RT controls in RT-qPCR to specifically control for residual DNA amplification in the samples. The publication cited for gametocyte quantification (https://doi:10.1371/journal.pone.0076316.) does not explain the use of male and female specific targets (PfMGET and CCp4) nor the use of in vitro RNA constructs. Please amend the correct citations for clarity about the used methods in the study. Since the Pf gametocyte positivity and median gametocyte density was quite high in mono-infections (91.8%/140 g/µL), it would be good to clarify the methodology to know if correct removal of residual DNA or the use of Intron spanning primers was performed. Additionally, for better clarity it could be mentioned that gametocytaemia is the sum of male and female gametocytes in the methods section (as specified in line 218 also).

**Response #5:** We appreciate the reviewer’s detailed feedback and have amended the methods section accordingly. We have now included the manufacturer’s instructions to support the RNA extraction procedure, and we explicitly referenced the manufacturer’s protocol (lines 133 - 135). To ensure accurate molecular quantification of gametocytes, residual DNA was removed by DNase enzyme digestion prior to RT-qPCR, since the *Pvs25* primers used are not intron-spanning. Non-RT controls were included in the RT-qPCR assays to specifically monitor residual DNA amplification as the reviewer rightly mentioned but we missed to include it in the original version which we now rectified. We clearly mentioned this in the methods section (lines 139 - 143). Furthermore, we have updated a citation to include studies that describe the use of male- and female-specific gametocyte markers (*PfMGET* and *CCp4*) (Meerstein-Kessel *et al.*, 2018) and removed the wrongly cited reference. Also, in vitro RNA constructs have been used in previous studies and reported there in the supplemental information (Andolina *et al.*, 2021), thereby we provided clarity on the methodological framework. To clarify, *P. falciparum* gametocyte density represents the sum of male and female gametocytes. There is no expected residual DNA contamination in estimating *P. falciparum* gametocytes since the primers we used were designed as intron spanning and therefore only amplify RNA transcripts. Therefore, *P. falciparum* gametocyte positivity and median gametocyte density that was quite high in mono-infections were the real findings in our study.

3. For sporozoite quantification you name a PCR assay (line 157). I suppose this should read qPCR, otherwise please amend the methods section and explain which PCR assay was used with the correct reference.

**Response #6:** We thank the reviewer for this comment. Sporozoite quantification was done by 18S based qPCR, and it is now clarified, reads as “qPCR” line 168.

4. It remains unclear whether sporozoite quantification was done on whole mosquitoes (as described in the methods line 153 vs day 12 sporozoite detection by 18s qPCR of salivary glands). For clarity, when extracting DNA from whole mosquitoes it is not really a sporozoite quantification, as oocysts might still be present in the midguts, especially when mosquitoes were not offered a second blood meal and you extract “relatively” early on day 12. When interested in sporozoites, the dissection of head/thorax from the abdomen is recommendable, to separate oocysts from sporozoites (although not salivary gland sporozoites exclusively). This should be clarified and explained in both methods and results and can be mentioned as limitation of the study. Otherwise, please explain the methods for salivary gland dissection in the methods section when you did so.

**Response #7:** Thank you for raising this important point. We clarify that sporozoite quantification in our study was performed using the whole mosquito collected at day 12 post-infection. We acknowledge the reviewer’s comment that the signal can also come from oocysts, especially for *P. falciparum*; *P. vivax* sporogony takes shorter time. The use of whole mosquito carcasses does indeed leave uncertainty about whether salivary gland sporozoites or oocyst sporozoites were detected. This is now mentioned as relevant limitations and future consideration in the Discussion section (lines 388 - 392).

Line 82: ...may affect their, instead of ther 177/178: use qPCR instead of PCR.

**Response #8:** The typographical errors were corrected on the respective lines.

**Reviewer #2**: L148-151: It is unclear how many mosquitoes per patient blood were fed on, given the mosquito infection prevalence might vary by mosquito numbers. How many mosquitoes remain post-day10 dissection?

**Response #9:** On average 100-120 mosquitoes were exposed per feeding experiment but only median of 30 (IQR: 30-32) randomly picked mosquitoes were dissected on day 7-10 and a median of 20 (IQR: 15-20) randomly picked mosquitoes processed on day 12 for sporozoite detection by qPCR. In direct membrane feeding assays, 20-50 are typically dissected (Bousema *et al.*, 2012) and the precision in the proportion of infected mosquitoes depends on both the number dissected and the number infected. The difference between exposed mosquitoes and dissected mosquitoes was not dictated by mosquito survivorship; instead, the number of dissected or examined mosquitoes was defined by the logistics of mosquito husbandry and midgut dissection and oocyst detection.

L152: Concern if 12 instead of 14 days post infection was sufficient for complete sporozoite development from oocysts inside the mosquito. This will affect sporozoite count in salivary gland.

**Response #10:** We thank the reviewer for raising this point. The same concern was raised by reviewer#1 and explained on **Response #7**. Whilst we expect that the majority sporozoites will have already migrated to the salivary glands (Andolina *et al.*, 2024; Evers *et al.*, 2026, under review), we agree that some oocysts will have remained unruptured by this day, especially for *P. falciparum*. Our findings are therefore not conclusive evidence that sporozoites have reached the salivary glands but sampling on day 12 does not compromise the assessment of sporozoite development, as all groups are equally subject to this biological variability. Thus, our choice of day 12 captures the period of peak salivary gland colonization, and the presence of unruptured oocysts beyond day 12 does not affect the validity of comparisons between experimental groups. Our prior evidence on *P. vivax* sporogony rather hint that it is a relatively rapid process and on day 12 post-feeding, oocysts rupture (Tadesse *et al.*, 2021). Acknowledging the reviewer’s comments, we have mentioned the limitation of sporozoite/oocyst detection in the revised Discussion section (lines 386 - 392).

L167-171: Concern that key covariates such as study site heterogeneity, age of patients, parasite density are not addressed or adjusted in the analyses when comparing infectivity.

**Response #11:** We thank the reviewer for this comment. To include key variables, we performed additional multivariable analyses adjusting for parasite density, age, mixed infection status, and study site. We fitted a series of generalized additive mixed models, with study site included as a fixed effect and participant ID as a random effect to account for repeated observations. The fully adjusted models (**Model 5 of S3 Table**) showed that there was no significant difference in mosquito infectivity between mono  and mixed-species infections for either *P. vivax* (AOR = 0.65, 95% CI: 0.29–1.48, *p*=0.307) or *P. falciparum* (AOR = 2.03, 95% CI: 0.93–4.42, *p*=0.076), consistent with our original findings. These additional analyses have now incorporated into the revised manuscript and presented in the methods (Lines 179 -183), result (lines 311 – 314), and Supplementary Table (**S3 Table**).

**Results**
**Reviewer #1:** The analysis makes sense to the reviewer, although he has limited experience in statistics. Results are well presented and figures are clear. Overall, the results presented in the study match the well-studied, basic concepts of human-to-mosquito transmission which is mediated mainly by gametocyte density in the human host. This is also nicely underlined by the present study. Indeed, the infection rates of mosquitoes are in all (mono Pf / Pv and mixed infections) quite high for DMFAs with no serum replacement conducted. Therefor this is a solid dataset, given the complexity of the experiments (DMFAs) this is very much appreciated by the reviewer!

**Minor comments results:**

1. Microscopy data is missing on parasite numbers (gametocytes and asexual parasites). It would be nice to compare gametocyte density and asexual parasite densities at the microscopic level with molecular quantification. Even only for mono-infections. If this data is available, it could be presented in the supplementary information.

**Response #12:** We thank the reviewer for raising this point. We have microscopy data for most of the mono infections; both asexual and sexual stage quantified separately that we didn’t include in the manuscript before. In response to this comment of the reviewer, we have compared the microscope quantification data for both asexual and sexual stages in the **Supplementary figure; S1 Fig**. There is strong correlation between the two methods for both *P. falciparum* and *P. vivax* though a weaker association was observed between *P. vivax* gametocyte density by microscopy and *Pvs25* transcripts copies by RT-qPCR. An important observation was that microscopy missed approximately half of the mixed infections that were detected by qPCR. This has implications in case management, related with differing treatment regimens for the two species in Ethiopia, chloroquine plus 14-day primaquine for *P. vivax* radical cure and artemether-lumefantrine plus single low dose primaquine for transmission blocking in *P. falciparum*. We have included this in the results (lines 193 - 196) and discussion sections (lines 333 - 341).

2. Table 2: Is it mean oocyst density or median oocyst density? Last row reads: “Mean oocysts density, median (IQR)”. Please specify.

**Response #13:** In our analyses, mean oocyst density was first calculated per feeding. The values presented in Table 2 represent the median of these mean oocyst densities across all feedings. We have now revised the table legend (line 264) to specify this more clearly, ensuring that readers understand that the summary statistics shown are the median (IQR) of the mean oocyst density values.

3. From the reviewer’s knowledge and the literature, many infected mosquitoes from natural malaria infections (or even most) only harbour 1-2 oocysts. However, in your results in table 2, under median oocyst density you say (5-32) Pv mono, (5-54) Pf mono and (4-53) mixed. Looking at this impressive overall number of infected guts (in the thousands), it seems very unlikely to the reviewer that you have not detected a single mosquito midgut with 1,2 or 3 oocysts. Please check this in your data.

**Response #14:** We thank the reviewer for this observation. We agree that in natural malaria infections, many mosquitoes harbour only 1–2 oocysts, and we also observe such low-density infections in our study which was masked by how we presented it. We have reviewed our data to examine the distribution of oocyst counts (histogram, see below). We have now added this detail in the supplemental information (**S2 Fig A-B**). Though the overall median of mean oocyst density was above four for all infection categories, we have seen non negligible number of mosquitoes per individuals that harbour 1-3 oocysts. As shown in **S2 Fig B**, we have limited the maximum number of oocysts per mosquito to 20 to show clearly the frequency of individuals harbouring few oocysts per midgut, (lines 258 – 259).

4. Table 3: Pv and Pf gametocyte density, please add NA or 0 (0/9), (0/5) for the specie that should not be detected in mono infections.

**Response #15:** We added NA for the species that should not appear in either of the mono infections.

Line 195: ...gametocyte density was higher in P. vivax mono-infections...

**Response #16:** At first “in” was missing between higher and *P. vivax* and now we added it. Now it reads as “gametocyte density was higher in *P. vivax* mono-infections….”, (line 213).

Line 204: Violin plot showing the median parasite instead of parasiste,

**Response #17:** The typographic error in line 204 corrected now. It reads as “parasite”, (line 223).

Reviewer #2: L175: The percentage is not equal with the number in parenthesis, 50.9% (220/511), please check and correct it.

**Response #18:** We thank the reviewer for pointing this out. Apologies for the typographical error. The correct value should be 43.1%. We have now corrected this in the revised manuscript to ensure accuracy (line 188)**.**

L178: Suggest present prevalence and infectivity data stratify by study sites as suppl data.

**Response #19:** We thank the reviewer for this suggestion in presenting the data stratified by site for prevalence and infectivity. Accordingly, we have shown the prevalence and infectivity of each infection category based on the sites in the table as supplementary (**S1 Table**).

L187-202: The total parasite and gametocyte densities were compared between mono and mixed Pf and Pv infections. But it is unclear how parasite/gametocyte densities were measured? By microscopy or qPCR? If by qPCR, need to describe the calculation of how to transform CT values into parasite or gametocyte/ul of blood using standard serial dilution and whether absolute or relative quantification was considered in the Method.

**Response #20:** Thank you for raising this important point. Parasite and gametocyte density were measured by both microscopy and qPCR (RT-qPCR) but the final comparison between mono and mixed infections was using molecular quantification since microscopy missed most of the mixed infections. Detailed procedures on how to transform CT values to parasite or gametocyte/uL of blood are explained in **Response #4** and to show the robustness of the molecular methods, we compared microscopy results with molecular quantification for mono infections as presented in **Response #12**.

L198-200: Check median and p-value as 2D seems to show overlapping median and range like that in 2B.

**Response #21:** Thank you for pointing this out. We re‑examined the data underlying **Fig. 2D**. The reported medians (163 gametocytes/µL for *P. falciparum* mono‑infections versus 49.5 gametocytes/µL for mixed‑species infections) are correct and were calculated from the full dataset. The interquartile ranges (median, 163 gametocytes/µL; IQR 40–720) versus (median 49.5 gametocytes/µL; IQR 5 – 704; Fig 2D), the Mann–Whitney U test comparing the two groups yielded a highly significant difference (*p*<0.001). This is not uncommon since the IQR demonstrates the spread in the data, not the precision of (differences between) medians.

L210-216: In Fig 3, why the unit are different e.g., panel A presents transcript copies/ul while panel B present gametocytes/ul? Likewise, inconsistence between 3A & B regarding Pv18s copies vs. parasite density.

**Response #22:** Thank you for raising this point. The difference in units between panels A and B reflects the distinct quantification approaches we used for *P. vivax* (**panel A**) and *P. falciparum* **(panel B).** This was also raised by another reviewer, and we explained it in **Response #4.**

Also, in panel B, it is not clear whether the parasitemia/gametocytema represents both species or Pf only. The statistical values in the figure are not readable and some gene names like Pvs25 needs to be italicized.

**Response #23:** We thank the reviewer for their detailed observations. To clarify, the parasitemia and gametocytemia values shown in panel B represent *P. falciparum* only. We have revised the figures to improve readability by adjusting the size of the embedded text and clearly indicated what species the panels refer to in the legend. In addition, gene names such as *Pvs25* and *Pv18S* have now been italicized in accordance with standard nomenclature in **Fig. 3**.

L218: how to calculate the sum of male & female gametocytes.

**Response #24:** The calculation of total *P. falciparum* gametocyte density was performed by summing the densities of male and female gametocytes, which were quantified separately. Specifically, gametocyte counts were derived from qRT-PCR assays targeting sex-specific transcripts: *PfMGET* for male gametocytes and *CCP4* for female gametocytes. Based on transcript-to-cell conversion factors, one gametocyte corresponds to approximately 9.8 transcripts of *PfMGET* and 4 transcripts of *CCP4* (Meerstein-Kessel *et al.*, 2018). Accordingly, we first converted transcript counts into gametocyte numbers for each sex independently and then added these values together to obtain the overall gametocyte density per µL. We have now added this steps to the method section, (lines 139 – 146). This approach has been used repeatedly in published articles (Andolina *et al.*, 2021; Rek *et al.*, 2022).

L227: “falciparum mono-infections this was 72.0% (108/150) …” remove “this” or rephrase the statement.

**Response #25:** The statement was rephrased and now reads as “The prevalence of infectious feeds was 75.4% (214/284) for *P. vivax* mono-infections, 72.0% (108/150) for *P. falciparum* mono-infections, and 76.6% (59/77) for *Pv–Pf* mixed infections”; (lines 248 – 252).

L225-228: Unclear how many patients were included in DMFA? all 511 or only gametocyte-positive patients? Was 14,884 the total mosquitoes being fed in DMFA? If so, it's ~30 mosquitoes per patient for all 511 patients in each feed, which is way too low given 30 were used for day7/10 oocyst dissection and remaining for day12 sporozoite assessment.

**Response #26:** A total of 511 patients were included in the DMFA (line 187). n total, 14,884 mosquitoes were dissected across the 511 feedings; this figure refers to the number dissected and does not represent the total mosquitoes offered for feeding. Between 100 and 120 mosquitoes were used per patient, giving an estimated 51,100 – 61,320 mosquitoes used for feeding in total. For each patient, approximately 30 mosquitoes were dissected at day 7/10 for oocyst assessment, while the remaining mosquitoes were maintained until day 12 for sporozoite detection and quantification. Although the number dissected per patient may appear limited, this approach was determined to be statistically sufficient and logistically feasible, as dissecting more than 30 mosquitoes per patient was time and resource demanding. Previous studies have proposed a minimum of 17 (Sawa *et al.*, 2013) or 20 dissected mosquitoes (Bousema *et al.*, 2006)**.**

L230: Unclear how many patient samples this derived from, all 284 mono-Pv? as there could be inter-patient variation in mosquito infectivity, would be important to show the range of the proportion of infected mosquito for each infection type.

**Response #27:** The analysis was derived from 284 *P. vivax* mono-infections. Infectiousness was defined as infecting at least one mosquito with at least one oocyst. We acknowledge that the proportion of infected mosquitoes varies across individuals due to inter-patient variation in mosquito infectivity. To address this, we have now included the range of infected mosquitoes across infection types to better illustrate**.** It reads as “the median proportion of infected mosquitoes among infectious feeds for *P. vivax* mono is 56.7% (IQR: 26.7 - 83.3), for *P. falciparum* is 64.8% (IQR: 34.4 - 81.3), and for mixed infection 63.3% (IQR: 30.0 – 78.1)” (lines 248 – 252).

L247-248: This is unclear. Should this be rewrite as among the 384 sporozoite-positive mosquitoes, how many were detected with Pv+Pf, Pv only, and Pf only. Also, should the denominator the same (384 or 386 mosquitoes)? Why the denominator for both species is 386, Pf is 90, and Pv is 134?

**Response #28:** We thank the reviewer for this helpful suggestion. This classification was originally based on separating individuals who transmitted only *P. vivax*, only *P. falciparum*, or both species simultaneously. Following the reviewer’s recommendation, we have now used the same denominator, corresponding to the total number of mosquitoes dissected from the 48 mixed Pv–Pf patients. In total, 51.2% (384/750) of the mosquitoes were infected from 48 feedings. Among them, 16.1% (62/384) were infected with *P. vivax* sporozoites only, 15.9% (61/384) with *P. falciparum* sporozoites only, and 68.0% (261/384) with both species. While the absolute numbers of infected mosquitoes remain unchanged, the proportions are now recalculated using the common denominator, thereby improving clarity and consistency. We now edited the sentence in line 275– 277.

Table 3: Again, why the denominator is different? should this be based on total # of mosquitoes fed on the 48 mixed Pv-Pf patients, and among the total, how many were Pv-sporozoite infected only, Pf-sporozoite infected only, and Pv/Pf-sporozoite infected.

**Response #29**: The table is now updated based on the **Response #28**.

Figures quality needs improved.

**Response #30:** We improved the quality of the figures as per the journal standards.

**Conclusions**

**Reviewer #1:** The conclusions of the present study are supported by the data presented. Minor limitations could be better described and conclusions for competition better discussed. (Comments below). The discussion is sound. The public health relevance is not addressed in the discussion, but probably this is not necessary, the reviewer has no clear opinion whether this is needed.

**Response #31:** Thank you for the feedback. Following the inclusion of the microscopy data and the discordance we observed compared to qPCR in identifying mixed species infections, we have added what the public health implication of this is in the revised discussion section of the manuscript (lines 332 -341).

**Minor comments conclusion/discussion**:

5. Your results indicate that gametocyte density is the most important determinant of transmissibility. Also, you show that for mono and mixed infections the prevalence of infectious feeds and the proportion of infected mosquitoes was very similar. How do you explain this, given the fact that in mixed infection your median gametocyte density is much lower compared to both mono infection gametocyte medians? I understand that you refer to per gametocyte infectivity, however, this concept is not clear as you do not explain what a gametocyte really is for Pv (how many copies/is one gametocyte?). Please elaborate/add clarity on this.

**Response #32:** Thank you for this thoughtful observation. We agree that further clarification is needed. Gametocyte density in this study was quantified by qRT‑PCR targeting gametocyte transcript (*Pvs25*), which measures transcript copies per volume rather than direct gametocyte cell counts. Conversion factors between transcript copies and gametocyte numbers are well characterized for *P. falciparum* but remain uncertain for *P. vivax*; therefore, our gametocyte estimates are relative and may not reflect absolute gametocyte counts. Consequently, the term “per‑gametocyte infectivity” is used operationally to denote mosquito infection probability per unit transcript. Similar mosquito infection prevalence in mono‑ and mixed infections despite lower median *Pvs25* transcripts in mixed infections could reflect:

(i) Mosquito infection probability increases non‑linearly with gametocyte density; low densities can still produce infections and large increases in density yield diminishing returns. This explains why similar mosquito infection can occur across different median transcript levels.
(ii) For *P. falciparum*, qRT‑PCR studies have quantified transcript copies per gametocyte (e.g., pfs25 ≈ 279 copies/gametocyte; male marker much lower), enabling conversion from transcripts to gametocytes; equivalent validated conversions for *P. vivax* are lacking, so *Pvs25* values are relative.
(iii) Transmission requires both sexes; shifts toward an optimal male:female ratio (or relatively more male fertility) can increase infectivity per female‑transcript unit. Measuring both male and female markers (sex‑specific qRT‑PCR) can clarify this.
(iv) Inter‑species facilitation / host factors: mixed infections may alter host immunity or parasite biology (e.g., gametocyte maturation or activation), increasing per‑gametocyte infectivity even at lower transcript abundance. Empirical reports document efficient transmission from mixed infections.

These alternatives cannot be resolved without validated *P. vivax* transcript to cell conversion standards, sex‑specific quantification, and additional investigations which we highlighted in the limitation and future direction section of the manuscript (lines 366 -371).

6. You said you did not find an indication for competition in the mosquito. However, literature shows that parasite development (EIP) in the mosquito is strongly linked to mosquito nutritional status (lipids, Aas eg from the mosquito blood meals). This has been confirmed in natural infections in DMFAs. It’s clear to the reviewer that the design of your study does not allow to study these details and this was probably also not the scope of your work. However, presumably competition in the mosquito is first between (any species of parasites) oocysts undergoing sporogony and the mosquito’s egg production and then finally visible at the sporozoite stage. Probably, this effect is also density related (many oocysts more competition than few). At the sporozoite stage, a potential advantage of one species over another would be measurable in terms of how many sporozoites are produced by either species (weighed by gametocyte density of each species fed to the mosquito). From your results it’s not clear whether you have compared sporozoite numbers of Pf and Pv in mixed infections (I am referring to Figure 2 in the supplementary), it seems you have quite a difference there in terms of copy numbers. So probably there is still evidence for competition. I guess this is the point where you would be able to conclude/quantify on a potential competition between parasite species within the mosquito. Maybe you can elaborate more on this finding and eventually suggest what further studies could do to better understand competition in the mosquito.

**Response #33:** Thank you for this insightful comment. We agree that competition within the mosquito is a complex process, and our study design was not intended to dissect the nutritional or physiological mechanisms underlying parasite development. As you rightly note, literature has shown that mosquito nutritional status (lipids, amino acids from blood meals) strongly influences parasite development and extrinsic incubation period (EIP). We didn’t compare the sporozoite numbers of Pf and Pv in mixed infections in now updated as **supplementary Figure; S4 Fig**. Rather, we compare each species sporozoite density separately between mosquitoes carrying single species and mixed species to explore if sporozoite count is high or low in the presence of either of the species in the mosquitoes. Sporozoite count in the mosquitoes was done based on 18S rRNA gene target by qPCR. Since the method of sporozoite 18S gene-based quantification was different between the species (standard difference), we were unable to compare between *P. vivax* and *P. falciparum* sporozoite density for mixed species infected mosquitoes but were able to determine whether co-infection with the alternative species (e.g. Pf) affected sporozoite densities of a *Plasmodium* species (e.g. Pv). Future work should combine a better method to quantify both species in the mosquitoes that can be converted to sporozoite numbers that would help us to compare between the two species in the mosquito, and we mentioned this the discussion section to better clarify limitations of our work (lines 392 – 398).

L342: Plasmodium in italics

**Response #34:** Done accordingly.

**Reviewer #2:** L26: Replace the phrase “…incompletely understood” to “… poorly understood”.

**Response #35:** This is now paraphrased based on the comment (line 24).

L36-37: The sentence ‘…Transmission to mosquitoes was high across infections and infection combinations…” needs to be clearer. What is the reference to say high as 56.3% infection rate in mosquitoes is not that high?

**Response #36:** We thank the reviewer for this helpful comment. The sentence was intended to indicate that the overall proportion of infectious feeds to mosquitoes was high across infection types (*P. vivax* mono, *P. falciparum* mono, and mixed *Pv-Pf* infections). As shown, among the 48 individuals carrying both species, 56.3% (27/48) transmitted both parasites. Importantly, some of the individuals also transmitted either *P. vivax* or *P. falciparum* to individual mosquitoes. We have now clarified the wording to better reflect that the term “high” refers to the overall proportion of infectious feeds across infection categories, rather than the absolute infection rate in mosquitoes (lines 34 -35).

L298: Typo “…may explain why especially”.

**Response #37:** We now corrected the typographical error; (line 331).

L300-306: It seems a conflicting idea. While the prevalence of Pv gametocyte is similar in mixed and mono infections, it is an overstatement that gametocyte production is efficient. This could just mean almost all Pv infections had gametocytes (typical asynchronous and early development features of Pv). On the other hand, Pv and Pf gametocyte densities were lower in mixed infections. The lower gametocyte densities seem to contradict the notion of efficient gametocyte production.

**Response #38:** Thank you for this careful point. We agree that prevalence and density are distinct metrics and that prevalence alone does not demonstrate “efficient” gametocyte production. In our data *P. vivax* gametocyte prevalence (the proportion of infections with detectable gametocyte transcripts) was similar in mono‑ and mixed‑species infections, while *P. vivax* gametocyte density (transcript copies per volume) was lower in mixed infections. We have revised the manuscript to remove the term “efficient production” and now state that similar prevalence with lower density is consistent with common gametocyte occurrence but reduced gametocyte biomass in mixed infections; this pattern may reflect within‑host interactions (competition or modulation) or differences in the timing/asynchrony of *P. vivax* gametocytogenesis. We have added a limitation noting that gametocyte measures are transcript‑based (*Pvs25* for *P. vivax*) and therefore reflect detection and relative abundance rather than absolute cell counts, and we recommend sex‑specific assays and validated *P. vivax* transcript to cell standards for future work (lines 381 -387).

L42, 315-323: The conclusion of “no competition” between Plasmodium species could be overinterpreted. Pf gametocyte prevalence and density are reduced in mixed infections and Pv asexual density is also reduced in mixed infections. This suggests perhaps some competition (or interaction) in the human host, even though gametocyte infectivity is similar in the mosquitoes.

**Response #39:** Thank you for this comment. We agree that the phrase “no competition” may overstate our findings. In our dataset, we observed reduced *P. falciparum* gametocyte prevalence and reduced density in mixed infections, which is consistent with within host interactions or competition for resources. At the same time, mosquito infection prevalence following membrane feeding was similar between mono‑ and mixed infections. These two observations can coexist if mixed infections alter the per‑gametocyte transmission efficiency (for example via sex‑ratio shifts, differences in transcript copy number per gametocyte, or host immune modulation), or if the relationship between gametocyte biomass and mosquito infection is non‑linear. We have therefore revised the Discussion to remove categorical language and now state that mixed infections show evidence of within‑host interaction while mosquito infectivity did not show clear competitive exclusion; we also added a limitation noting that gametocyte density was measured as transcript abundance (*Pvs25*) rather than absolute gametocyte counts and that sex‑specific quantification is needed to resolve mechanisms lines 381 – 387).

**Summary and General Comments**

Use this section to provide overall comments, discuss strengths/weaknesses of the study, novelty, significance, general execution and scholarship. You may also include additional comments for the author, including concerns about dual publication, research ethics, or publication ethics. If requesting major revision, please articulate the new experiments that are needed.

**Reviewer #1:** In this study, Chali et al. investigated how mixed infections of P. falciparum and P. vivax differ in terms of parasite densities and if mixed vs mono infections affect transmission of gametocytes of both parasite species to mosquitoes. The main outcome of the study is that although parasite densities differ between mono and mixed infections, no direct consequence of competition between both species when infecting the same mosquito could be detected. Therefore, the authors conclude that in presence of both species, transmission potential is not altered. The objectives of the study are clearly defined and the study appropriately designed to address the stated objectives with minor weaknesses to determine competition in the mosquito at the sporozoite stage (commented under conclusions). Overall, results support the drawn conclusions and are well discussed. The study population is clearly described, sample sizes are sufficient and statistical analysis is correct, according to the limited knowledge of the reviewer. This is a well written, well conducted and solid piece of work on a poorly investigated field in malaria research with the focus on transmission of mixed Plasmodium infections. Therefore, it presents valuable knowledge to the interested scientific community. Congratulations for the good work, the reviewer really enjoyed the reading!

**Reviewer #3: Overview**

The manuscript addresses an important knowledge gap whether mixed infections with Plasmodium falciparum and *P. vivax* alter transmission to mosquitoes. A key finding includes no evidence for interspecies competition within mosquitoes, which has implications for effective malaria control and elimination strategy, especially where both parasites occur in sympatry. It is a well written manuscript with clear research gaps. The methodology both in terms of design and analytical procedures employed is appropriate, and discussion and conclusions fitting to the data generated in the study.

**I highlight few issues for consideration:**

• Please, clarity on how the present study differs from that of Balasubramanian et al. 2020 (ref 32), which also showed efficient mixed-species transmission in Cambodia.

**Response #40:** We thank the reviewer for raising this point. Balasubramanian *et al.* (2020) demonstrated that mixed *P. vivax–P. falciparum* infections in Cambodia can be transmitted efficiently to mosquitoes. Their study showed that *P. vivax* was more readily transmitted than *P. falciparum*, and that multiple parasite clones from mixed infections could be passed on to mosquitoes, thereby maintaining parasite diversity in the vector population. Our study differs in several important ways. First, we analyzed a much larger cohort that included mono-*P. vivax*, mono-*P. falciparum*, and mixed infections, which allowed direct comparison of transmission efficiency across infection types. Second, we quantified gametocyte densities using sex-specific qRT-PCR assays, providing more precise measures of parasite contribution to transmission. Third, we extended the analysis beyond oocyst detection to include sporozoite quantification, offering a more complete picture of transmission potential. Taken together, while Balasubramanian *et al*. highlighted the ability of mixed infections to transmit and preserve parasite diversity, our study expands these findings by providing a broader dataset, more detailed gametocyte quantification, and sporozoite-level outcomes, thereby offering deeper insights into the epidemiological role of mixed-species infections.

• How were the titers of the parasites in the exposed blood before and after feeding mosquitoes in the DMFAs? This could be done in freshly engorged mosquitoes as well and better appreciate titer differences imposed by experimental handling and mosquito ingestion.

**Response #41**: We appreciate this comment of the reviewer. We assumed that the number of parasites ingested by mosquitoes is adequately reflected by their density in the blood sample used for feeding. We previously indicated that gametocyte concentrations of *P. falciparum* measured in blood and in the mosquito blood meal immediately after engorging and were strongly correlated (Meibalan *et al.*, 2021). We agree with the reviewer that this could have been a good addition to the data considering different species evaluated in this study. Acknowledging the important comment, we have now included this in the future perspectives of the discussion section for future studies to evaluate it (lines 385 – 387).

• The authors should clarify if there is any data on the level of arboviral pathogens in the study areas, increasingly recognized as important contributors of febrile illness other than malaria.

**Response #42:** We thank the reviewer for highlighting the relevance of arboviral pathogens in the study areas. We agree that this is an area of increasing importance. Our study population consisted of patients with confirmed malaria, and our analyses were restricted to malaria parasites. We did not include screening for other causes of febrile illness as this is outside the scope of the study.

• The Pf quantification uses NF54 ring-stage parasites as opposed to Pv that uses plasmid constructs. This has implication in the reporting units for parasite densities between the species (parasites/µL vs copies/µL). Any potential discrepancies and justification that may affect cross-species density comparisons should be provided.

**Response #43:** We appreciate the reviewer’s observation regarding the differences in quantification approaches between *P. falciparum* and *P. vivax*. Our main aim was to compare the parasite itself when it exists alone or in mixed infection with another species, to evaluate if its infectiousness was affected by the presence of the other species, rather than direct comparison of gametocyte density and parasite density across species with different quantification approach. This was raised in the previous comments and explained in **Response#4.**

• Clarity on DMFA timing is required. Pv oocysts were assessed at day 7, Pf at day 10 (L147-148) and mixed infections assessed on day 10 only. Possibility exists that by day 10 some Pv oocysts may have ruptured and could be missed.

**Response #44:** We thank the reviewer for raising this point regarding DMFA timing. We acknowledge that by day 10, some *P. vivax* oocysts may have ruptured and released sporozoites to the salivary glands. However, the slower developing *P. falciparum* oocysts require additional time to mature, and assessing at day 10 ensures their detection, thereby confirming infectivity regardless of species at the oocyst stage. Of note, oocyst sheets may still be detected on day 10 in case of ruptured oocysts. We did not observe these. This gives us confidence that our assessments are robust, further supported that bias would only be introduced if the rate of rupture of *P. vivax* oocysts would differ for Pv-Pf infected vs *P. vivax*-only infected mosquitoes. This is highly unlikely, especially since *P. falciparum* oocyst densities are typically lower than *P. vivax* and unlikely to result in nutritional shortages for developing *P. vivax* oocysts. To further analyse species-specific transmission in mixed infections, we additionally performed sporozoite stage detection and differentiation. At this stage, no parasite will be missed due to timing, and we can reliably determine which species is transmitted. This approach allowed us to balance the biological differences in parasite development while ensuring accurate assessment of infectivity outcomes**.**

• It is stated in the results section that sporozoite detection was done on day 12 on 750 mosquitoes from 48 DMFAs using mixed-infection blood. I think this rationale should be captured in the methods section.

**Response #45:** We thank the reviewer for this observation. It was now updated in Methods section (lines 157 – 163).

• The GAMM specification specifies poisson distribution but can be susceptible to overdispersion (L168-171). With proportional mosquito infection data from feeding assays, this can be substantial. It may be better to test other distributions (e.g. nb, quasi poisson, etc) and not only poisson to allow for selection of the best fit model supported by model residuals.

**Response #46:** Thank you for the comment. We agree that Poisson models can be susceptible to overdispersion and may not be appropriate for proportional mosquito infection data from feeding assays. However, in the submitted Methods section, the GAMM was specified with a binomial distribution, not a Poisson distribution. This was because the outcome was mosquito infection status/proportion, modelled as the number infected out of the total number dissected. The model included log10-transformed gametocyte density and infection type as fixed effects, with a random intercept for individual ID to account for repeated measures. To avoid any ambiguity, we have revised the text to explicitly state that the response variable was the number of infected mosquitoes out of the total dissected, and that a binomial distribution was used; lines 178 – 182).

• Another limitation worth acknowledging in the extrapolation of DMFA results to natural transmission. Important behavioral components of transmission relating to host-seeking, probing and partial feeding and whether feeding occurs selectively on certain individuals, would be missed via DMFAs.

**Response #47:** We thank the reviewer for highlighting this important limitation. We agree that DMFAs do not fully represent natural transmission. Behavioural components of vectors on transmission such as host-seeking, probing, partial feeding, or selective feeding on certain individuals are indeed missed in DMFA settings. However, these factors do not affect the primary objective of our study, which was to compare parasite and gametocyte density and mosquito infectivity between mono-infections and mixed *P. vivax–P. falciparum* infections. Since we used the same laboratory-colonized mosquito colony across all infection types, the absence of natural behavioural variation does not bias our comparative analyses.

**Minor comments**

• L68: Insert ‘to’ between it and complete (L68) and a between as and highly (L78)
• L82: Correct spelling of ther to their
• L104: Correct, P .vivax

• L204: Correct spelling parasiste” and “gametcoyte”
• L298: Correct spelling “expecially”,
• L300: “wassimilarly”

**Response #48:** All minor comments and typographical errors corrected accordingly.

• The authors should cross check the text (L191-194) and table 2 for potential inconsistencies in data reporting: Pf gametocyte positivity in mono-infections was 91.8% (135/147), but table 1 indicates N=150 for Pf mono-infections.

**Response #49:** We thank the reviewer for carefully cross-checking the data presentation. The apparent discrepancy arises because gametocyte data were not available for three patients with *P. falciparum* mono-infections. Thus, while Table 1 reports a total of 150 *P. falciparum* mono-infections, the denominator for gametocyte positivity in the results section is 147, reflecting only those patients with available gametocyte data. We have now clarified this in the legend of table to avoid confusion; line 262.

**References**

Andolina, C., Graumans, W., Guelbeogo, M., van Gemert, G. J., Ramijth, J., Harouna, S.*, et al.* (2024). **Quantification of sporozoite expelling by Anopheles mosquitoes infected with laboratory and naturally circulating P. falciparum gametocytes**. *Elife,* **12**. <https://doi:10.7554/eLife.90989>

Andolina, C., Rek, J. C., Briggs, J., Okoth, J., Musiime, A., Ramjith, J.*, et al.* (2021). **Sources of persistent malaria transmission in a setting with effective malaria control in eastern Uganda: a longitudinal, observational cohort study**. *Lancet Infect Dis,* **21**(11), 1568–1578. <https://doi:10.1016/S1473-3099(21)00072-4>

Bousema, J. T., Schneider, P., Gouagna, L. C., Drakeley, C. J., Tostmann, A., Houben, R.*, et al.* (2006). **Moderate effect of artemisinin-based combination therapy on transmission of Plasmodium falciparum**. *J Infect Dis,* **193**(8), 1151–1159. <https://doi:10.1086/503051>

Bousema, T., Dinglasan, R. R., Morlais, I., Gouagna, L. C., van Warmerdam, T., Awono-Ambene, P. H.*, et al.* (2012). **Mosquito feeding assays to determine the infectiousness of naturally infected Plasmodium falciparum gametocyte carriers**. *PloS one,* **7**(8), e42821. <https://doi:10.1371/journal.pone.0042821>

Evers, F., Gemert, G., Lanke, K., Graumans, W., Andolina, C., Mordmüller, B.*, et al.* (2026, under review). **Quantifying sporozoite inoculum dynamics with the SpitGrid reveals temporal**

**decay and behavioural determinants of Plasmodium transmission**. *bioRxiv,* **Under Review**. <https://doi>: <https://doi.org/10.64898/2026.01.06.697895>

Meerstein-Kessel, L., Andolina, C., Carrio, E., Mahamar, A., Sawa, P., Diawara, H.*, et al.* (2018). **A multiplex assay for the sensitive detection and quantification of male and female Plasmodium falciparum gametocytes**. *Malar J,* **17**(1), 441. <https://doi:10.1186/s12936-018-2584-y>

Meibalan, E., Barry, A., Gibbins, M. P., Awandu, S., Meerstein-Kessel, L., Achcar, F.*, et al.* (2021). **Plasmodium falciparum Gametocyte Density and Infectivity in Peripheral Blood and Skin Tissue of Naturally Infected Parasite Carriers in Burkina Faso**. *J Infect Dis,* **223**(10), 1822–1830. <https://doi:10.1093/infdis/jiz680>

Rek, J., Blanken, S. L., Okoth, J., Ayo, D., Onyige, I., Musasizi, E.*, et al.* (2022). **Asymptomatic School-Aged Children Are Important Drivers of Malaria Transmission in a High Endemicity Setting in Uganda**. *J Infect Dis,* **226**(4), 708–713. <https://doi:10.1093/infdis/jiac169>

Sawa, P., Shekalaghe, S. A., Drakeley, C. J., Sutherland, C. J., Mweresa, C. K., Baidjoe, A. Y.*, et al.* (2013). **Malaria transmission after artemether-lumefantrine and dihydroartemisinin-piperaquine: a randomized trial**. *J Infect Dis,* **207**(11), 1637–1645. <https://doi:10.1093/infdis/jit077>

Tadesse, F. G., Ashine, T., Teka, H., Esayas, E., Messenger, L. A., Chali, W.*, et al.* (2021). **Anopheles stephensi Mosquitoes as Vectors of Plasmodium vivax and falciparum, Horn of Africa, 2019**. *Emerg Infect Dis,* **27**(2), 603–607. <https://doi:10.3201/eid2702.200019>

Tadesse, F. G., Chali, W., Ejigu, L. A., Abdo, M., Kassa, F. A., Bulto, M. G.*, et al.* (2026). **Parasite and gametocyte dynamics and infectivity to mosquitoes during recurrent Plasmodium vivax infections: A longitudinal study from Ethiopia**. *J Infect,* **92**(6), 106756. <https://doi:10.1016/j.jinf.2026.106756>

Tadesse, F. G., Slater, H. C., Chali, W., Teelen, K., Lanke, K., Belachew, M.*, et al.* (2018). **The relative contribution of symptomatic and asymptomatic Plasmodium vivax and Plasmodium falciparum infections to the infectious reservoir in a low-endemic setting in Ethiopia**. *Clinical infectious diseases,* **66**(12), 1883–1891.
