# Supplementary material for: Plasmodium vivax and Plasmodium falciparum mixed infections in human and mosquito hosts: The impact of multi-species infection on parasite densities and transmission to mosquitoes
Source: PLoS Negl Trop Dis. 2026 Jul 24;20(7):e0014148. doi: 10.1371/journal.pntd.0014148 (PMC13446731; doi:10.1371/journal.pntd.0014148)
Supplement: Peer review history

# Peer review: Decision Letter 0

**DOI:** 10.1371/journal.pntd.0014148.r001

**Authors:** Claudia Ida Brodskyn, Rhoel Ramos Dinglasan

15 Apr 2026

PNTD-D-26-00504

Plasmodium vivax and Plasmodium falciparum mixed infections in human and mosquito hosts: the impact of multi-species infection on parasite densities and transmission to mosquitoes

PLOS Neglected Tropical Diseases

Dear Dr. Tadesse,

Thank you for submitting your manuscript to PLOS Neglected Tropical Diseases. After careful consideration, we feel that it has merit but does not fully meet PLOS Neglected Tropical Diseases's publication criteria as it currently stands. Therefore, we invite you to submit a revised version of the manuscript that addresses the points raised during the review process.

Please submit your revised manuscript within by Jun 14 2026 11:59PM. If you will need more time than this to complete your revisions, please reply to this message or contact the journal office at plosntds@plos.org. When you're ready to submit your revision, log on to https://www.editorialmanager.com/pntd/ and select the 'Submissions Needing Revision' folder to locate your manuscript file.

Please include the following items when submitting your revised manuscript:

* A letter that responds to each point raised by the editor and reviewer(s). You should upload this letter as a separate file labeled 'Response to Reviewers'. This file does not need to include responses to any formatting updates and technical items listed in the 'Journal Requirements' section below.

* A marked-up copy of your manuscript that highlights changes made to the original version. You should upload this as a separate file labeled 'Revised Manuscript with Track Changes'.

* An unmarked version of your revised paper without tracked changes. You should upload this as a separate file labeled 'Manuscript'.

If you would like to make changes to your financial disclosure, competing interests statement, or data availability statement, please make these updates within the submission form at the time of resubmission. Guidelines for resubmitting your figure files are available below the reviewer comments at the end of this letter.

We look forward to receiving your revised manuscript.

Kind regards,

Rhoel Ramos Dinglasan

Academic Editor

PLOS Neglected Tropical Diseases

Claudia Brodskyn

Section Editor

PLOS Neglected Tropical Diseases

Shaden Kamhawi

co-Editor-in-Chief

PLOS Neglected Tropical Diseases

orcid.org/0000-0003-4304-636XX

Paul Brindley

co-Editor-in-Chief

PLOS Neglected Tropical Diseases

orcid.org/0000-0003-1765-0002

**Journal Requirements:**

1) We do not publish any copyright or trademark symbols that usually accompany proprietary names, eg ©,  ®, or TM  (e.g. next to drug or reagent names). Therefore please remove all instances of trademark/copyright symbols throughout the text, including:

- ® on page: 8.

2) We notice that your supplementary Tables are included in the manuscript file. Please remove them and upload them with the file type 'Supporting Information'. Please ensure that each Supporting Information file has a legend listed in the manuscript after the references list.

3) Figure 1: please (a) provide a direct link to the base layer of the map (i.e., the country or region border shape) and ensure this is also included in the figure legend; and (b) provide a link to the terms of use / license information for the base layer image or shapefile. We cannot publish proprietary or copyrighted maps (e.g. Google Maps, Mapquest) and the terms of use for your map base layer must be compatible with our CC BY 4.0 license.

Note: if you created the map in a software program like R or ArcGIS, please locate and indicate the source of the basemap shapefile onto which data has been plotted.

If your map was obtained from a copyrighted source please amend the figure so that the base map used is from an openly available source. Alternatively, please provide explicit written permission from the copyright holder granting you the right to publish the material under our CC BY 4.0 license.

If you are unsure whether you can use a map or not, please do reach out and we will be able to help you. The following websites are good examples of where you can source open access or public domain maps:

* U.S. Geological Survey (USGS) - All maps are in the public domain. (http://www.usgs.gov)

* PlaniGlobe - All maps are published under a Creative Commons license so please cite “PlaniGlobe, http://www.planiglobe.com, CC BY 2.0” in the image credit after the caption. (http://www.planiglobe.com/?lang=enl)

* Natural Earth - All maps are public domain. (http://www.naturalearthdata.com/about/terms-of-use/)

**Reviewers' Comments:**

Reviewer's Responses to Questions

**Key Review Criteria Required for Acceptance?**

As you describe the new analyses required for acceptance, please consider the following:

**Methods**

-Are the objectives of the study clearly articulated with a clear testable hypothesis stated?

-Is the study design appropriate to address the stated objectives?

-Is the population clearly described and appropriate for the hypothesis being tested?

-Is the sample size sufficient to ensure adequate power to address the hypothesis being tested?

-Were correct statistical analysis used to support conclusions?

-Are there concerns about ethical or regulatory requirements being met?

Reviewer #1: Minor comments methodology:

1. Molecular quantification of parasites is a main outcome variable in the study for both gametocytes and asexual parasites. Undoubtedly, this is done at a sufficient quality here. Still, reconstitution of standard curves for asexual parasites should span at least 6 magnitudes (eg 10^8-10^3 as done for the gametocytes), with identical dilution series for Pf and Pv. This would improve the quality of parasite quantification. It is logic, that for P. vivax plasmids are used. However, if the relation between copy numbers and parasite densities taking the copy number of the 18s gene in P. vivax is not taken into consideration, this is not really a parasite quantification rather an estimation based on a quantification of copy numbers. This should be mentioned in the methods section. One could think of mention this as a limitation of the study in the discussion.

2. Taking the importance of correct molecular quantification for the present study, RNA extraction should be well supported by citations or the manufacturers protocol should be mentioned at least. Most importantly, the procedure for removal of residual DNA should be explained to assure that you are really quantifying gametocytes since pvs25 is also detectable as DNA not only RNA transcripts. It is recommendable to mention not only the use of negative controls (as you do correctly) but also the use of Non-RT controls in RT-qPCR to specifically control for residual DNA amplification in the samples. The publication cited for gametocyte quantification (https://doi:10.1371/journal.pone.0076316.) does not explain the use of male and female specific targets (PfMGET and CCp4) nor the use of in vitro RNA constructs. Please amend the correct citations for clarity about the used methods in the study. Since the Pf gametocyte positivity and median gametocyte density was quite high in mono-infections (91.8%/140 g/µL), it would be good to clarify the methodology to know if correct removal of residual DNA or the use of Intron spanning primers was performed. Additionally, for better clarity it could be mentioned that gametocytemia is the sum of male and female gametocytes in the methods section (as specified in line 218 also).

3. For sporozoite quantification you name a PCR assay (line 157). I suppose this should read qPCR, otherwise please amend the methods section and explain which PCR assay was used with the correct reference.

4. It remains unclear weather sporozoite quantification was done on whole mosquitoes (as described in the methods line 153 vs day 12 sporozoite detection by 18s qPCR of salivary glands). For clarity, when extracting DNA from whole mosquitoes it is not really a sporozoite quantification, as oocysts might still be present in the midguts, especially when mosquitoes were not offered a second blood meal and you extract “relatively” early on day 12. When interested in sporozoites, the dissection of head/thorax from the abdomen is recommendable, to separate oocysts from sporozoites (although not salivary gland sporozoites exclusively). This should be clarified and explained in both methods and results and can be mentioned as limitation of the study. Otherwise, please explain the methods for salivary gland dissection in the methods section when you did so.

Line 82: ...may affect their instead of ther

177/178: use qPCR instad of PCR

Reviewer #2: L148-151: It is unclear how many mosquitoes per patient blood were fed on, given the mosquito infection prevalence might vary by mosquito numbers. How many mosquitoes remain post-day10 dissection?

L152: Concern if 12 instead of 14 days post infection was sufficient for complete sporozoite development from oocysts inside the mosquito. This will affect sporozoite count in salivary gland.

L167-171: Concern that key covariates such as study site heterogeneity, age of patients, parasite density are not addressed or adjusted in the analyses when comparing infectivity.

Reviewer #3: See comments to authors

**********

**Results**

-Does the analysis presented match the analysis plan?

-Are the results clearly and completely presented?

-Are the figures (Tables, Images) of sufficient quality for clarity?

Reviewer #1: The analysis makes sense to the reviewer, although he has limited experience in statistics. Results are well presented and figures are clear. Overall, the results presented in the study match the well studied, basic concepts of human-to-mosquito transmission which is mediated mainly by gametocyte density in the human host. This is also nicely underlined by the present study. Indeed, the infection rates of mosquitoes are in all (mono Pf / Pv and mixed infections) quite high for DMFAs with no serum replacement conducted. Therefor this is a solid dataset, given the complexity of the experiments (DMFAs) this is very much appreciated by the reviewer!

Minor comments results:

1. Microscopy data is missing on parasite numbers (gametocytes and asexual parasites). It would be nice to compare gametocyte density and asexual parasite densities at the microscopic level with molecular quantification. Even only for mono-infections. If this data is available, it could be presented in the supplementary information.

2. Table 2: Is it mean oocyst density or median oocyst density? Last row reads: “Mean oocysts density, median (IQR)”. Please specify.

3. From the reviewer’s knowledge and the literature, many infected mosquitoes from natural malaria infections (or even most) only harbour 1-2 oocysts. However, in your results in table 2, under median oocyst density you say (5-32) Pv mono, (5-54) Pf mono and (4-53) mixed. Looking at this impressive overall number of infected guts (in the thousands), it seems very unlikely to the reviewer that you have not detected a single mosquito midgut with 1,2 or 3 oocysts. Please check this in your data.

4. Table 3: Pv and Pf gametocyte density, please add NA or 0 (0/9), (0/5) for the specie that should not be detected in mono infections.

Line 195: ...gametocyte density was higher in P. vivax mono-infections...

Line 204: Violin plot showing the median parasite instead of parasiste

Reviewer #2: L175: The percentage is not equal with the number in parenthesis, 50.9% (220/511), please check and correct it.

L178: Suggest present prevalence and infectivity data stratify be study sites as suppl data.

L187-202: The total parasite and gametocyte densities were compared between mono and mixed Pf and Pv infections. But it is unclear how parasite/gametocyte densities were measured? By microscopy or qPCR? If by qPCR, need to describe the calculation of how to transform CT values into parasite or gametocyte/ul of blood using standard serial dilution and whether absolute or relative quantification was considered in the Method.

L198-200: Check median and p-value as 2D seems to show overlapping median and range like that in 2B.

L210-216: In Fig 3, why the unit are different e.g., panel A presents transcript copies/ul while panel B present gametocytes/ul? Likewise, inconsistence between 3A & B regarding Pv18s copies vs. parasite density.

Also, in panel B, it is not clear whether the parasitemia/gametocytema represents both species or Pf only. The statistical values in the figure are not readable and some gene names like Pvs25 needs to be italicized.

L218: how to calculate the sum of male & female gametocytes.

L227: “falciparum mono-infections this was 72.0% (108/150)…” remove “this” or rephrase the statement.

L225-228: Unclear how many patients were included in DMFA? all 511 or only gametocyte-positive patients? Was 14,884 the total mosquitoes being fed in DMFA? If so, it's ~30 mosquitoes per patient for all 511 patients in each feed, which is way too low given 30 were used for day7/10 oocyst dissection and remaining for day12 sporozoite assessment.

L230: Unclear how many patient samples this derived from, all 284 mono-Pv? as there could be inter-patient variation in mosquito infectivity, would be important to show the range of the proportion of infected mosquito for each infection type.

L247-248: This is unclear. Should this be rewrite as among the 384 sporozoite-positive mosquitoes, how many were detected with Pv+Pf, Pv only, and Pf only. Also, should the denominator the same (384 or 386 mosquitoes)? Why the denominator for both species is 386, Pf is 90, and Pv is 134?

Table 3: Again, why the denominator is different? should this be based on total # of mosquitoes fed on the 48 mixed Pv-Pf patients, and among the total, how many were Pv-sporozoite infected only, Pf-sporozoite infected only, and Pv/Pf-sporozoite infected.

Figures quality needs improved.

Reviewer #3: See comments to authors

**********

**Conclusions**

-Are the conclusions supported by the data presented?

-Are the limitations of analysis clearly described?

-Do the authors discuss how these data can be helpful to advance our understanding of the topic under study?

-Is public health relevance addressed?

Reviewer #1: The conclusions of the present study are supported by the data presented. Minor limitations could be better described and conclusions for competition better discussed. (Comments below). The discussion is sound.

the public health relevance is not addressed in the discussion, but probably this is not necessary, the reviewer has no clear opinion whether this is needed.

Minor comments conclusion/discussion:

5. Your results indicate that gametocyte density is the most important determinant of transmissibility. Also, you show that for mono and mixed infections the prevalence of infectious feeds and the proportion of infected mosquitoes was very similar. How do you explain this, given the fact that in mixed infection your median gametocyte density is much lower compared to both mono infection gametocyte medians? I understand that you refer to per gametocyte infectivity, however, this concept is not clear as you do not explain what a gametocyte really is for Pv (how many copies/is one gametocyte?). Please elaborate/add clarity on this.

6. You said you did not find an indication for competition in the mosquito. However, literature shows that parasite development (EIP) in the mosquito is strongly linked to mosquito nutritional status (lipids, Aas eg from the mosquito blood meals). This has been confirmed in natural infections in DMFAs. It’s clear to the reviewer that the design of your study does not allow to study these details and this was probably also not the scope of your work. However, presumably competition in the mosquito is first between (any species of parasites) oocysts undergoing sporogony and the mosquito’s egg production and then finally visible at the sporozoite stage. Probably, this effect is also density related (many oocysts more competition than few). At the sporozoite stage, a potential advantage of one species over another would be measurable in terms of how many sporozoites are produced by either species (weighed by gametocyte density of each species fed to the mosquito). From your results it’s not clear whether you have compared sporozoite numbers of Pf and Pv in mixed infections (I am referring to Figure 2 in the supplementary), it seems you have quite a difference there in terms of copy numbers. So probably there is still evidence for competition. I guess this is the point where you would be able to conclude/quantify on a potential competition between parasite species within the mosquito. Maybe you can elaborate more on this finding and eventually suggest what further studies could do to better understand competition in the mosquito.

342: Plasmodium in italics

Reviewer #2: L26: Replace the phrase “…incompletely understood” to “… poorly understood”.

L36-37: The sentence ‘…Transmission to mosquitoes was high across infections and infection combinations…” needs to be clearer. What is the reference to say high as 56.3% infection rate in mosquitoes is not that high?

L298: Typo “…may explain why especially”.

L300-306: It seems a conflicting idea. While the prevalence of Pv gametocyte is similar in mixed and mono infections, it is an overstatement that gametocyte production is efficient. This could just mean almost all Pv infections had gametocytes (typical asynchronous and early development features of Pv). On the other hand, Pv and Pf gametocyte densities were lower in mixed infections. The lower gametocyte densities seem to contradict the notion of efficient gametocyte production.

L42, 315-323: The conclusion of “no competition” between Plasmodium species could be overinterpreted. Pf gametocyte prevalence and density are reduced in mixed infections and Pv asexual density is also reduced in mixed infections. This suggests perhaps some competition (or interaction) in the human host, even though gametocyte infectivity is similar in the mosquitoes.

Reviewer #3: See comments to authors

**********

**Editorial and Data Presentation Modifications?**

Use this section for editorial suggestions as well as relatively minor modifications of existing data that would enhance clarity. If the only modifications needed are minor and/or editorial, you may wish to recommend “Minor Revision” or “Accept”.

Reviewer #1: The reviewer recommends the reviewed manuscript with "Minor Revisions".

Reviewer #2: (No Response)

Reviewer #3: See comments to authors

**********

**Summary and General Comments**

Use this section to provide overall comments, discuss strengths/weaknesses of the study, novelty, significance, general execution and scholarship. You may also include additional comments for the author, including concerns about dual publication, research ethics, or publication ethics. If requesting major revision, please articulate the new experiments that are needed.

Reviewer #1: In this study, Chali et al. investigated how mixed infections of P. falciparum and P. vivax differ in terms of parasite densities and if mixed vs mono infections affect transmission of gametocytes of both parasite species to mosquitoes.

The main outcome of the study is that although parasite densities differ between mono and mixed infections, no direct consequence of competition between both species when infecting the same mosquito could be detected. Therefor, the authors conclude that in presence of both species, transmission potential is not altered.

The objectives of the study are clearly defined and the study appropriately designed to address the stated objectives with minor weaknesses to determine competition in the mosquito at the sporozoite stage (commented under conclusions). Overall, results support the drawn conclusions and are well discussed. The study population is clearly described, sample sizes are sufficient and statistical analysis is correct, according to the limited knowledge of the reviewer. This is a well written, well conducted and solid piece of work on a poorly investigated field in malaria research with the focus on transmission of mixed Plasmodium infections. Therefor, it presents valuable knowledge to the interested scientific community. Congratulations for the good work, the reviewer really enjoyed the reading!

Reviewer #2: (No Response)

Reviewer #3: Overview

The manuscript addresses an important knowledge gap whether mixed infections with Plasmodium falciparum and P. vivax alter transmission to mosquitoes. A key finding includes no evidence for interspecies competition within mosquitoes, which has implications for effective malaria control and elimination strategy, especially where both parasites occur in sympatry. It is a well written manuscript with clear research gaps. The methodology both in terms of design and analytical procedures employed is appropriate, and discussion and conclusions fitting to the data generated in the study.

I highlight few issues for consideration:

• Please, clarity on how the present study differs from that of Balasubramanian et al. 2020 (ref 32), which also showed efficient mixed-species transmission in Cambodia.

• How were the titers of the parasites in the exposed blood before and after feeding mosquitoes in the DMFAs? This could be done in freshly engorged mosquitoes as will, and better appreciate titer differences imposed by experimental handling and mosquito ingestion.

• The authors should clarify if there is any data on the level of arboviral pathogens in the study areas, increasingly recognized as important contributors of febrile illness other than malaria.

• The Pf quantification uses NF54 ring-stage parasites as opposed to Pv that uses plasmid constructs. This has implication in the reporting units for parasite densities between the species (parasites/µL vs copies/µL). Any potential discrepancies and justification that may affect cross-species density comparisons should be provided.

• Clarity on DMFA timing is required. Pv oocysts were assessed at day 7, Pf at day 10 (L147-148) and mixed infections assessed on day 10 only. Possibility exists that by day 10 some Pv oocysts may have ruptured and could be missed.

• It is stated in the results section that sporozoite detection was done on day 12 on 750 mosquitoes from 48 DMFAs using mixed-infection blood. I think this rationale should be captured in the methods section.

• The GAMM specification specifies poisson distribution but can be susceptible to overdispersion (L168-171). With proportional mosquito infection data from feeding assays, this can be substantial. It may be better to test other distributions (e.g. nb, quasi poisson, etc) and not only poisson to allow for selection of the best fit model supported by model residuals.

• Another limitation worth acknowledging in the extrapolation of DMFA results to natural transmission. Important behavioral components of transmission relating to host-seeking, probing and partial feeding and whether feeding occurs selectively on certain individuals, would be missed via DMFAs.

Minor comments

• L68: Insert ‘to’ between it and complete (L68) and a between as and highly (L78)

• L82: Correct spelling of ther to their

• L104: Correct, P .vivax

• L204: Correct spelling parasiste” and “gametcoyte”

• L298: Correct spelling “expecially”,

• L300: “wassimilarly”

• The authors should cross check the text (L191-194) and table 2 for potential inconsistencies in data reporting: Pf gametocyte positivity in mono-infections was 91.8% (135/147), but table 1 indicates N=150 for Pf mono-infections.

**********

PLOS authors have the option to publish the peer review history of their article (what does this mean?). If published, this will include your full peer review and any attached files.

If you choose “no”, your identity will remain anonymous but your review may still be made public.

**Do you want your identity to be public for this peer review?**  For information about this choice, including consent withdrawal, please see our Privacy Policy.

Reviewer #1: No

Reviewer #2: No

Reviewer #3: No

[NOTE: If reviewer comments were submitted as an attachment file, they will be attached to this email and accessible via the submission site. Please log into your account, locate the manuscript record, and check for the action link "View Attachments". If this link does not appear, there are no attachment files.]

**Figure resubmission:**  While revising your submission, we strongly recommend that you use PLOS’s NAAS tool (https://ngplosjournals.pagemajik.ai/artanalysis) to test your figure files. NAAS can convert your figure files to the TIFF file type and meet basic requirements (such as print size, resolution), or provide you with a report on issues that do not meet our requirements and that NAAS cannot fix.

After uploading your figures to PLOS’s NAAS tool - https://ngplosjournals.pagemajik.ai/artanalysis, NAAS will process the files provided and display the results in the "Uploaded Files" section of the page as the processing is complete. If the uploaded figures meet our requirements (or NAAS is able to fix the files to meet our requirements), the figure will be marked as "fixed" above. If NAAS is unable to fix the files, a red "failed" label will appear above. When NAAS has confirmed that the figure files meet our requirements, please download the file via the download option, and include these NAAS processed figure files when submitting your revised manuscript.

**Reproducibility:**

To enhance the reproducibility of your results, we recommend that authors of applicable studies deposit laboratory protocols in protocols.io, where a protocol can be assigned its own identifier (DOI) such that it can be cited independently in the future. Additionally, PLOS ONE offers an option to publish peer-reviewed clinical study protocols. Read more information on sharing protocols at https://plos.org/protocols?utm_medium=editorial-email&utm_source=authorletters&utm_campaign=protocols

Attachment Submitted filename: Review of PNTD-D-26-00504.docx

# Author comment: Author response to Decision Letter 1

**DOI:** 10.1371/journal.pntd.0014148.r002

16 Jun 2026

Attachment Submitted filename: Response_Review of PNTD-D-26-00504.docx

# Peer review: Decision Letter 1

**DOI:** 10.1371/journal.pntd.0014148.r003

**Authors:** Claudia Ida Brodskyn, Rhoel Ramos Dinglasan

12 Jul 2026

Dear Dr Tadesse,

We are pleased to inform you that your manuscript 'Plasmodium vivax and Plasmodium falciparum mixed infections in human and mosquito hosts: the impact of multi-species infection on parasite densities and transmission to mosquitoes' has been provisionally accepted for publication in PLOS Neglected Tropical Diseases.

Before your manuscript can be formally accepted you will need to complete some formatting changes, which you will receive in a follow up email. A member of our team will be in touch with a set of requests.

Please note that your manuscript will not be scheduled for publication until you have made the required changes, so a swift response is appreciated.

IMPORTANT: The editorial review process is now complete. PLOS will only permit corrections to spelling, formatting or significant scientific errors from this point onwards. Requests for major changes, or any which affect the scientific understanding of your work, will cause delays to the publication date of your manuscript.

Should you, your institution's press office or the journal office choose to press release your paper, you will automatically be opted out of early publication. We ask that you notify us now if you or your institution is planning to press release the article. All press must be co-ordinated with PLOS.

Thank you again for supporting Open Access publishing; we are looking forward to publishing your work in PLOS Neglected Tropical Diseases.

Best regards,

Rhoel Ramos Dinglasan

Academic Editor

PLOS Neglected Tropical Diseases

Claudia Brodskyn

Section Editor

PLOS Neglected Tropical Diseases

Shaden Kamhawi

co-Editor-in-Chief

PLOS Neglected Tropical Diseases

orcid.org/0000-0003-4304-636XX

Paul Brindley

co-Editor-in-Chief

PLOS Neglected Tropical Diseases

orcid.org/0000-0003-1765-0002

***********************************************************

Reviewer's Responses to Questions

**Key Review Criteria Required for Acceptance?**

As you describe the new analyses required for acceptance, please consider the following:

**Methods**

-Are the objectives of the study clearly articulated with a clear testable hypothesis stated?

-Is the study design appropriate to address the stated objectives?

-Is the population clearly described and appropriate for the hypothesis being tested?

-Is the sample size sufficient to ensure adequate power to address the hypothesis being tested?

-Were correct statistical analysis used to support conclusions?

-Are there concerns about ethical or regulatory requirements being met?

Reviewer #1: (No Response)

Reviewer #3: All queries raised have been satisfactorily addressed

**********

**Results**

-Does the analysis presented match the analysis plan?

-Are the results clearly and completely presented?

-Are the figures (Tables, Images) of sufficient quality for clarity?

Reviewer #1: 266: qPCR analysis of salivary glands > qPCR analysis of homogenized whole mosquitoes

Reviewer #3: OK

**********

**Conclusions**

-Are the conclusions supported by the data presented?

-Are the limitations of analysis clearly described?

-Do the authors discuss how these data can be helpful to advance our understanding of the topic under study?

-Is public health relevance addressed?

Reviewer #1: 336: strengethening > strengthening

Reviewer #3: OK

**********

**Editorial and Data Presentation Modifications?**

Use this section for editorial suggestions as well as relatively minor modifications of existing data that would enhance clarity. If the only modifications needed are minor and/or editorial, you may wish to recommend “Minor Revision” or “Accept”.

Reviewer #1: (No Response)

Reviewer #3: (No Response)

**********

**Summary and General Comments**

Use this section to provide overall comments, discuss strengths/weaknesses of the study, novelty, significance, general execution and scholarship. You may also include additional comments for the author, including concerns about dual publication, research ethics, or publication ethics. If requesting major revision, please articulate the new experiments that are needed.

Reviewer #1: The authors have responded to raised points adequately. No further comments.

Reviewer #3: All queries raised have been satisfactorily addressed

**********

PLOS authors have the option to publish the peer review history of their article (what does this mean?). If published, this will include your full peer review and any attached files.

If you choose “no”, your identity will remain anonymous but your review may still be made public.

**Do you want your identity to be public for this peer review?**  For information about this choice, including consent withdrawal, please see our Privacy Policy.

Reviewer #1: No

Reviewer #3: No

# Editor report: Acceptance letter

**DOI:** 10.1371/journal.pntd.0014148.r004

**Authors:** Claudia Ida Brodskyn, Rhoel Ramos Dinglasan

Dear Dr Tadesse,

We are delighted to inform you that your manuscript, "Plasmodium vivax and Plasmodium falciparum mixed infections in human and mosquito hosts: the impact of multi-species infection on parasite densities and transmission to mosquitoes," has been formally accepted for publication in PLOS Neglected Tropical Diseases.

We have now passed your article onto the PLOS Production Department who will complete the rest of the publication process. All authors will receive a confirmation email upon publication.

The corresponding author will soon be receiving a typeset proof for review, to ensure errors have not been introduced during production. Please review the PDF proof of your manuscript carefully, as this is the last chance to correct any scientific or type-setting errors. Please note that major changes, or those which affect the scientific understanding of the work, will likely cause delays to the publication date of your manuscript. Note: Proofs for Front Matter articles (Editorial, Viewpoint, Symposium, Review, etc...) are generated on a different schedule and may not be made available as quickly.

Soon after your final files are uploaded, the early version of your manuscript will be published online unless you opted out of this process. The date of the early version will be your article's publication date. The final article will be published to the same URL, and all versions of the paper will be accessible to readers.

For Research Articles, you will receive an invoice from PLOS for your publication fee after your manuscript has reached the completed accept phase. If you receive an email requesting payment before acceptance or for any other service, this may be a phishing scheme. Learn how to identify phishing emails and protect your accounts at https://explore.plos.org/phishing.

Thank you again for supporting open-access publishing; we are looking forward to publishing your work in PLOS Neglected Tropical Diseases.

Best regards,

Shaden Kamhawi

co-Editor-in-Chief

PLOS Neglected Tropical Diseases

Paul Brindley

co-Editor-in-Chief

PLOS Neglected Tropical Diseases
